# TRIM59 suppresses mitochondrial-associated apoptosis to facilitate progression in papillary renal cell carcinoma via the ACAT1-cardiolipin pathway

**DOI:** 10.1038/s41419-025-07913-5

**Published:** 2025-08-11

**Authors:** Haiyan Weng, Jiaxin Zhong, Ruiyi Yang, Beinan Han, Qiuchen Kong, Yimin Zhang, Wei Zhuang, Jingyi Wang, Hai Hu, Xiaorong Lin

**Affiliations:** 1 Department of Oncology, Sun Yat-sen Memorial Hospital, Sun Yat-sen University, Guangzhou, Guangdong Province China; 2 Diagnosis and Treatment Center of Breast Diseases, Shantou Central Hospital, Shantou, Guangdong Province China; 3 Clinical Research Center, Shantou Central Hospital, Shantou, Guangdong Province China; 4 Department of Pharmacy, Women and Children’s Hospital, School of Medicine, Xiamen University, Xiamen, Fujian Province China; 5 Department of Otolaryngology-Head and Neck Surgery, Sun Yat-sen Memorial Hospital, Sun Yat-sen University, Guangzhou, Guangdong Province China; 6 Breast Cancer Center, Zhejiang Cancer Hospital, Hangzhou Institute of Medicine, Chinese Academy of Sciences, Hangzhou, Zhejiang Province China

**Keywords:** Apoptosis, Prognostic markers

## Abstract

Papillary renal cell carcinoma (pRCC) is a challenging renal cell carcinoma subtype with poor prognosis and limited treatment options due to the lack of reliable biomarkers. The tripartite motif (TRIM) protein family is involved in various cellular processes, including oncogenesis. Among these, TRIM59 has emerged as a potential oncogene in multiple cancers; however, its role in pRCC progression remains unclear. Here, by using RNA sequencing data from The Cancer Genome Atlas (TCGA) and LASSO Cox regression analysis, we developed a prognostic model based on TRIM family genes for pRCC, with RiskScore demonstrating potential as a prognostic biomarker. Through the comparison of overall survival (OS) and progression-free survival (PFS), we identified TRIM59 as the primary research target. TRIM59 was markedly overexpressed in pRCC tissues, and correlated with poor OS. Functional studies showed that TRIM59 knockdown inhibited pRCC cell proliferation and induced mitochondrial-related apoptosis both in vitro and in vivo. Mechanistically, TRIM59 facilitated K27- and K63-linked ubiquitination and degradation of Acetyl-CoA Acetyltransferase 1 (ACAT1) at lysine 174 (K174), a critical enzyme in mitochondrial lipid metabolism. This disruption of lipid homeostasis in clear cell renal carcinoma (pRCC), particularly in mitochondrial cardiolipin metabolism, inhibited mitochondria-dependent apoptosis and, consequently, enhanced tumorigenesis. These findings suggest TRIM59 as a biomarker and potential therapeutic target, supporting precision oncology strategies for pRCC treatment.

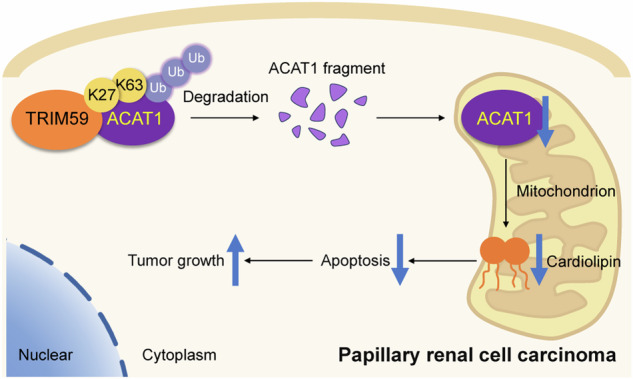

## Introduction

Renal cell carcinoma (RCC), often termed a “silent killer”, has an insidious onset and displays significant heterogeneity with a poor prognosis. Each year, approximately 400,000 new cases of RCC are reported worldwide, making it a major malignancy in the genitourinary system [1]. RCC originates from the epithelial cells of the renal parenchyma and urinary tubules and is broadly categorized into three subtypes: clear cell RCC (ccRCC), accounting for 65-70% of cases; papillary RCC (pRCC), comprising 15–20%; and chromophobe RCC (chRCC), representing 5–7% [2]. Multifocality is common in RCC, with pRCC often presenting as multifocal [3]. While ccRCC is the most prevalent subtype, nearly 40% of multifocal RCCs exhibit papillary features [4]. Due to the absence of specific molecular markers and distinguishable imaging or pathological characteristics, early diagnosis of pRCC is challenging, leading to many cases being identified at advanced or metastatic stages. In metastatic scenarios, pRCC outcomes are generally worse than those of ccRCC [5], highlighting the need for reliable biomarkers to improve clinical diagnosis, treatment, and prognosis for pRCC. Notably, renal cancer is a significant metabolic disease, with metabolic remodeling playing a crucial role in carcinogenesis [6], though the specific metabolic patterns and regulatory mechanisms in pRCC remain undocumented.

The tripartite motif (TRIM) protein family includes more than 80 members involved in diverse biological functions, such as morphogenesis [7], cell cycle regulation [8], cell signaling [9], transcriptional regulation [10], DNA repair [11], and protein quality control [12]. Over the past decade, studies have shown that TRIM proteins possess a structure comprising RING finger, B-box, and coiled-coil domains [13]. The presence of a RING domain indicates that TRIM proteins can function as E3 ubiquitin ligases [14]. In cancer biology, abnormal TRIM protein expression is closely linked to gene transcription regulation, cell proliferation, and apoptosis, influencing tumor growth [15]. However, the role and mechanism of TRIM proteins in pRCC progression remain unclear. Similar to other TRIM proteins, TRIM59 contains a tripartite motif and exhibits E3 ubiquitin ligase activity [16]. Previous studies have shown that TRIM59 is upregulated in various malignancies, including breast [17], pancreatic [18], and ovarian cancers [19]. Furthermore, it correlates with advanced clinical stages and lower survival rates. Mechanistically, TRIM59 promoted hepatocellular carcinoma growth by modulating the PPM1B/CDKs signaling pathway, affecting the G1/S phase of the cell cycle [20]. Additionally, TRIM59 facilitated gastric carcinogenesis by accelerating the ubiquitination and degradation of the p53 protein [21]. TRIM59 has also been implicated in promoting breast cancer metastasis via autophagic degradation through PDCD10 [22]. However, TRIM59’s role remains unexplored in pRCC.

In this study, we utilized datasets from public databases to establish a comprehensive risk prediction model based on TRIM family genes and to construct a prognostic nomogram for pRCC patients. Through immunohistochemical analysis of tissue microarrays, we assessed TRIM protein expression in normal kidney and pRCC tissues and evaluated the correlation between TRIM expression and patient survival outcomes. Our findings suggest that TRIM59 is a potential biomarker for pRCC. Functionally, TRIM59 knockdown inhibited pRCC cell proliferation and apoptosis in vitro and in vivo. Mechanistically, TRIM59 interacted with ACAT1, promoting its ubiquitin-mediated degradation, which regulated cardiolipin expression to remodel lipid metabolism and influence pRCC cell apoptosis. Overall, our work presents a novel approach for developing personalized therapies and offers new insights into pRCC management.

## Results

### Alterative expression of TRIM family correlates to clinical outcome of papillary renal carcinoma

**Fig. 1:**
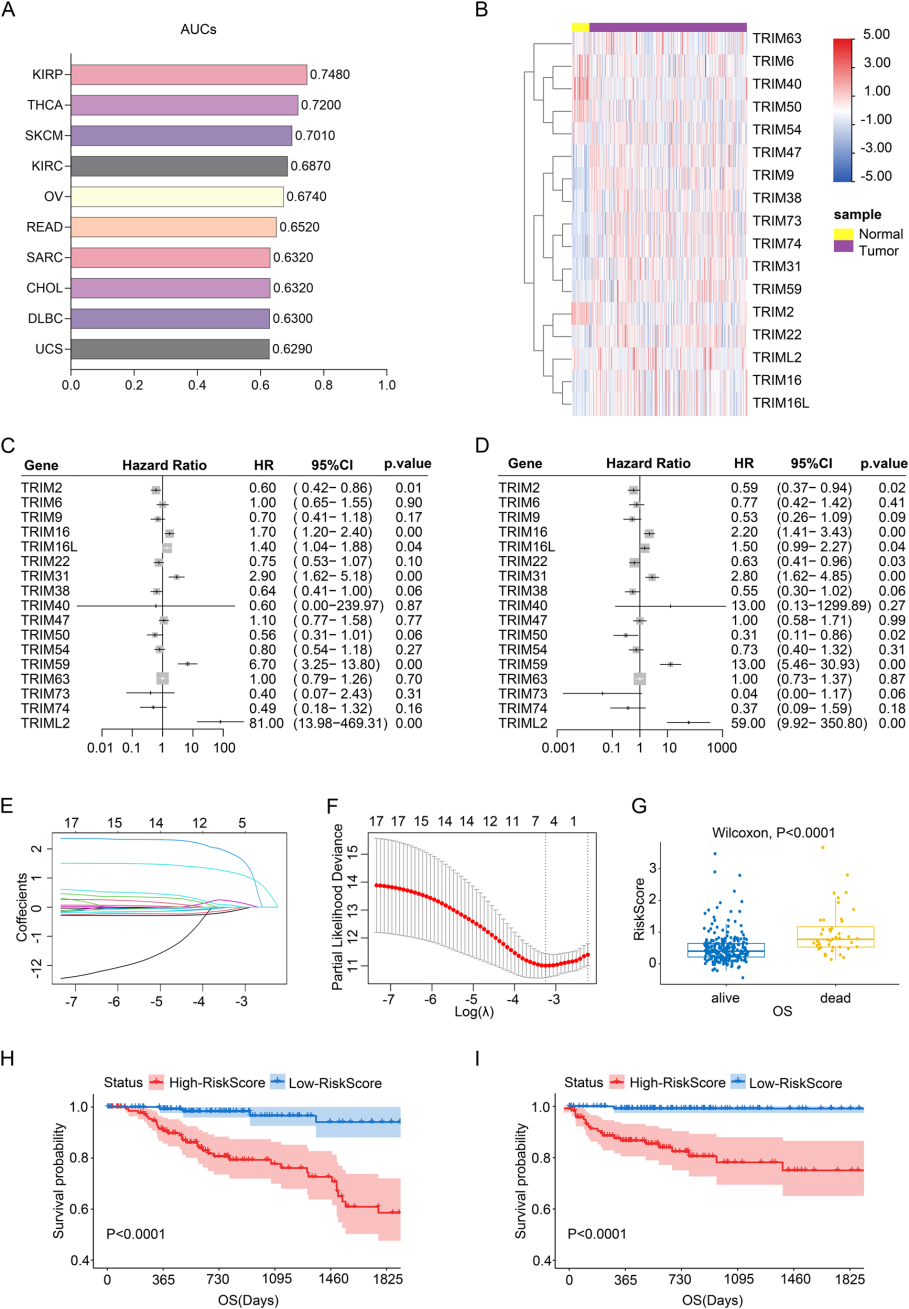
Alterative expression of TRIM family correlates to clinical outcome of papillary renal carcinoma. **A** Top ten AUC values of TRIM model in multiple tumors from TCGA database. **B** Heatmap showed the differential expression of TRIM family comparing the tumor tissues with relevant normal tissues in KIRP from TCGA database. **C** Forest plot showed the impact of TRIM family gene expression levels on overall survival (OS). **D** Forest plot showed the impact of TRIM family gene expression levels on progression-free survival (PFS). **E** LASSO regression analysis chart (10 fold cross validation, optimal λ = −3.2). **F** Cross validation of adjustment parameter selection in proportional risk model. **G** Comparison of RiskScore by OS Status in KIRP Patients (Wilcoxon Rank-Sum Test). **H** Survival curve plot of the impact of RiskScore expression on OS in the TCGA-KIRP dataset. **I** Survival curve plot of the effect of RiskScore expression on PFS in the TCGA-KIRP dataset.

We initially obtained TRIM family gene expression data for 33 tumor types from TCGA and applied LASSO regression for dimensionality reduction, alongside area under the curve (AUC) scoring. Papillary renal carcinoma yielded the highest AUC value, prompting further analysis of this subtype (Fig. 1A, Figure S1A). The interactions among differentially expressed TRIM family members (DEMs) at the protein level were depicted in the protein-protein interaction (PPI) network (Figure S1B), while Figure S1C illustrated the chromosomal distribution of copy number variations (CNVs) across 23 pairs of human chromosomes. Additionally, we highlighted the association between the TRIM family and Hazard Ratio (HR) based on overall survival (OS) and progression-free survival (PFS) across cancers (Figure S1D, E). Patients expressing *TRIM6*, *TRIM9*, *TRIM16*, *TRIM38*, *TRIM47*, *TRIM59* and *TRIML2* exhibited elevated HR for OS and PFS. In tumors such as adrenocortical carcinoma (ACC), kidney chromophobe (KICH), kidney papillary cell carcinoma (KIRP), and lower-grade glioma (LGG), TRIM gene expression was positively correlated with HR for both OS and PFS.

The TCGA dataset for papillary renal carcinoma included 287 tumor samples and 32 normal tissues, identifying 17 differentially expressed TRIM genes, with 13 upregulated and 4 downregulated in tumor tissues (Fig. 1B). To evaluate the impact of TRIM genes on OS and PFS in papillary renal carcinoma, we used the KM-plotter database. *TRIM16*, *TRIM16L*, *TRIM31*, *TRIM59*, and *TRIML2* were identified as significant risk factors for OS (*P* < 0.05, Fig. 1C), while *TRIM16*, *TRIM31*, *TRIM59*, and *TRIML2* also emerged as risk factors for PFS (*P* < 0.05, Fig. 1D). Among these, *TRIM59* and *TRIML2* exhibited the most pronounced effects on both OS and PFS.

To refine our analysis further, we performed LASSO Cox regression using the R package glmnet, integrating TRIM gene expression and OS data (Fig. 1E). As λ decreased, more independent variable coefficients approached zero. A 10-fold cross-validation was employed to develop the model, with confidence intervals for each λ (Fig. 1F). The resulting RiskScore equation was: RiskScore = exp.(TRIM38) × -0.0634 + exp.(TRIM31) × 0.0538 + exp.(TRIM16) × -0.1908 + exp.(TRIM2) × -0.1233 + exp.(TRIM59) × 1.1742 + exp.(TRIML2) × 1.7887. The classification of RiskScore was statistically significant (*P* < 0.001) (Fig. 1G), with high-risk samples associated with decreased OS (Fig. 1H) and PFS (Fig. 1I).

### RiskScore of TRIM family predicts prognosis and drug sensitivity in papillary renal carcinoma

**Fig. 2:**
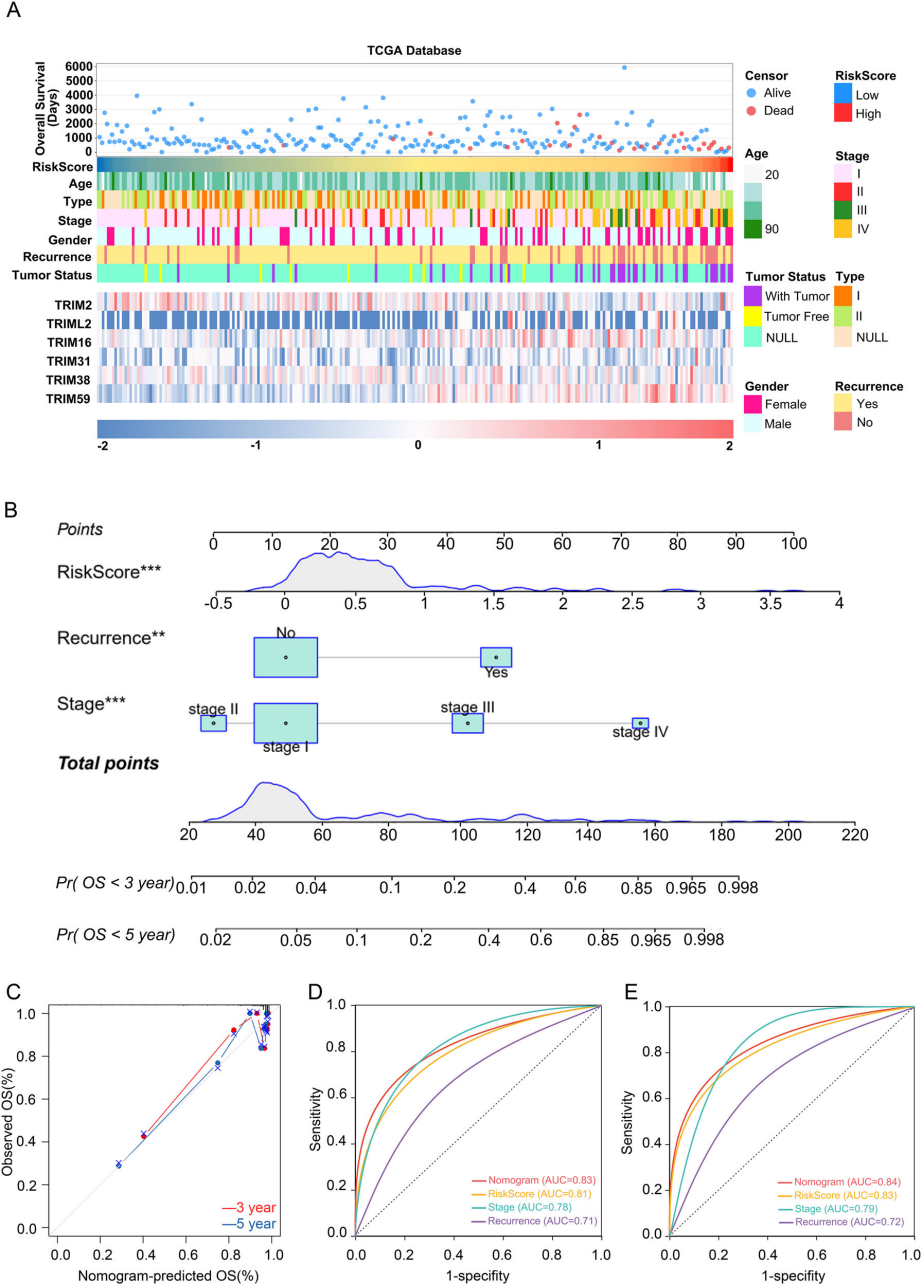
RiskScore of TRIM family predicts prognosis and drug sensitivity in papillary renal carcinoma. **A** The landscape of RiskScore-related clinicopathological features of papillary renal cell carcinoma in TCGA database. **B** Nomogram based on RiskScore, recurrence, and stage. **C** Calibration curve of nomogram. **D** Nomogram and ROC curve of single clinical features (3 years). **E** Nomogram and single ROC curve of clinical characteristics (5 years). **P < 0.01 and ***P < 0.001.

The TNM staging is strongly associated with outcome prediction, treatment options and tumor-specific mortality in clinical care [23]. We found that *TRIM2*, *TRIML2*, *TRIM16*, *TRIM38*, *TRIM59*, and RiskScore were significantly expressed across different T stages, suggesting their potential as diagnostic biomarkers for papillary renal cancer staging (Figure S2A). Additionally, the expression levels of *TRIML2*, *TRIM16*, and RiskScore varied significantly across clinical stages of papillary renal cancer, with advanced stages showing higher RiskScore (Figure S2B). Consistent with this, the linkage heat map of papillary renal carcinoma risk variables indicated that patients with high RiskScore often presented poorer prognostic factors, including higher mortality, advanced stages (III-IV), active tumor presence, female gender, and recurrence (Fig. 2A). A higher RiskScore was associated with poorer outcomes and increased mortality. Univariate and multivariate Cox regression analyses identified RiskScore (*P* < 0.001), recurrence (*P* < 0.001), and clinical stage (*P* < 0.001) as significant independent risk factors for OS (*P* < 0.01) (Table 1). Based on multivariate Cox regression results, a nomogram was constructed incorporating key clinical characteristics, including RiskScore, recurrence status, and stage (Fig. 2B). The 45° line on the calibration plot indicated optimal predictive performance, showing that the nomogram accurately predicted 3-year and 5-year survival outcomes (Fig. 2C). Additionally, the nomogram demonstrated a higher AUC at 3 and 5 years compared to nomograms based on individual clinical features (Fig. 2D, E).

**Table 1:** Univariate and multivariate COX regression analysis of the clinical features and RiskScore.

Variables	Univariable analysis				Multivariable analysis			
	HR	95% CI of HR		*P*	HR	95% CI of HR		*P*
		Lower	Upper			Lower	Upper	
RiskScore	4.571	3.017	6.925	7.61E-13	2.490	1.531	4.049	2.37E-04
Age	1.382	0.642	2.976	0.408				
Recurrence	11.475	4.889	26.929	2.07E-08	3.984	1.567	10.129	3.69E-03
Stage	3.129	2.212	4.424	1.11E-10	2.210	1.463	3.339	1.66E-04
Gender	0.880	0.375	2.065	0.769				

We then used the PharmacoDB database (https://pharmacodb.pmgenomics.ca/) to identify drugs associated with the TRIM gene family, selecting anticancer medications with a *P*-value < 0.05 and an absolute correlation coefficient > 0.1. Notably, *TRIM2* expression was positively correlated with sensitivity to Dabrafenib and Vemurafenib (*P* < 0.001), while *TRIM38* was positively correlated with sensitivity to Cediranib and Motesanib (*P* < 0.001). Conversely, elevated *TRIM59* expression was linked to reduced susceptibility to Vemurafenib (*P* < 0.001) (Figure S2C). These findings underscore that a combined-feature nomogram provided a more precise prediction of recurrence in patients with papillary renal cancer, aiding clinicians in decision-making and therapy customization.

### TRIM59 is the predominant risk factor of papillary renal carcinoma

**Fig. 3:**
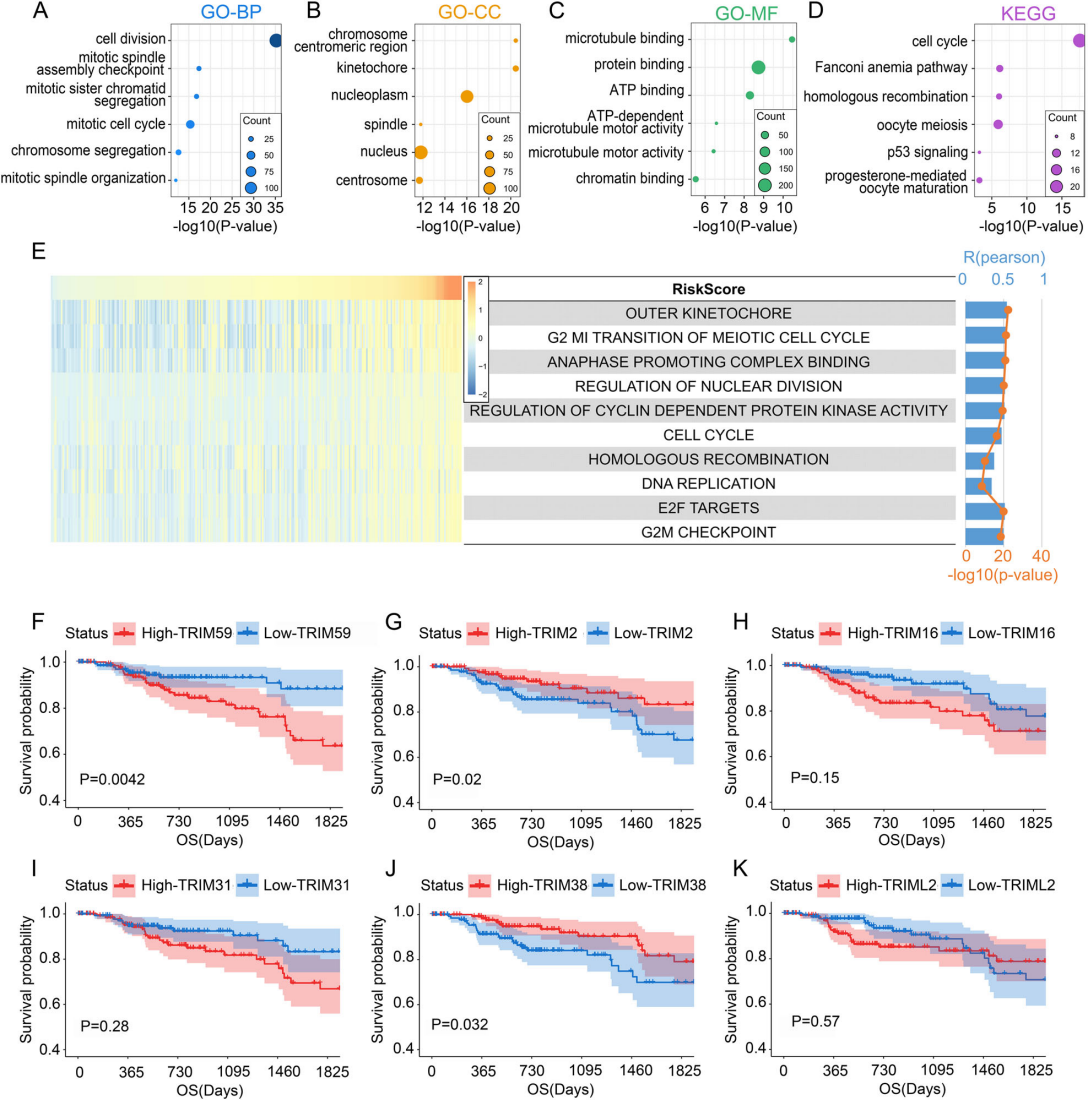
TRIM59 is the predominant risk factor of papillary renal carcinoma. **A**–**C** Biological processes (BP), cellular components (CC), and molecular functions (MF) were mostly related to RiskScore in TCGA database. **D** Kyoto Encyclopedia of Genes and Genomes (KEGG) pathway analysis of RiskScore in TCGA database. **E** Pearson correlation between RiskScore and KEGG. The width of the band represented the R-value. The color of the band represented the *P*-value. The correlation was tested by Pearson correlation analysis. **F**–**K** Survival curve of high- and low-TRIM59/TRIM2/TRIM16/TRIM31/TRIM38/TRIML2 groups.

To investigate the biological functions associated with RiskScore, Pearson correlation analysis was performed on TCGA data (*P* < 0.05) to identify genes most closely related to RiskScore. These gene sets were analyzed through Gene Ontology (GO) and KEGG pathway enrichment analyses. The biological processes most strongly associated with RiskScore included cell division, mitotic checkpoints, and chromosome segregation (Fig. 3A). Cellular components primarily linked to RiskScore were the chromosome, spindle apparatus, and nucleus (Fig. 3B), while molecular functions involved microtubule binding, protein binding, and ATP binding (Fig. 3C). Key pathways included the cell cycle, Fanconi anemia pathway, and p53 signaling pathway (Fig. 3D). Results from GSEA (Figure S3A) showed that RiskScore genes were enriched in processes such as cytoskeletal dynamics, G2/M checkpoints, and mitotic spindle organization. GSVA analysis (Fig. 3E) further validated these enrichment findings.

Among the six genes in the RiskScore, KM-plotter survival curves for 5-year OS analysis indicated that *TRIM59*, *TRIM2*, and *TRIM38* were significantly associated with OS in papillary renal carcinoma patients, with *P*-values of 0.0042, 0.02, and 0.032, respectively (Fig. 3F–K). However, other contributors, including *TRIM16* (*P* = 0.15), *TRIM31* (*P* = 0.28), and *TRIML2* (*P* = 0.57), did not significantly correlate with OS. Given the impact on OS, the biological function of *TRIM59* in pRCC captured our attention.

### TRIM59 suppresses the mitochondria related apoptosis of papillary renal cell carcinoma

**Fig. 4:**
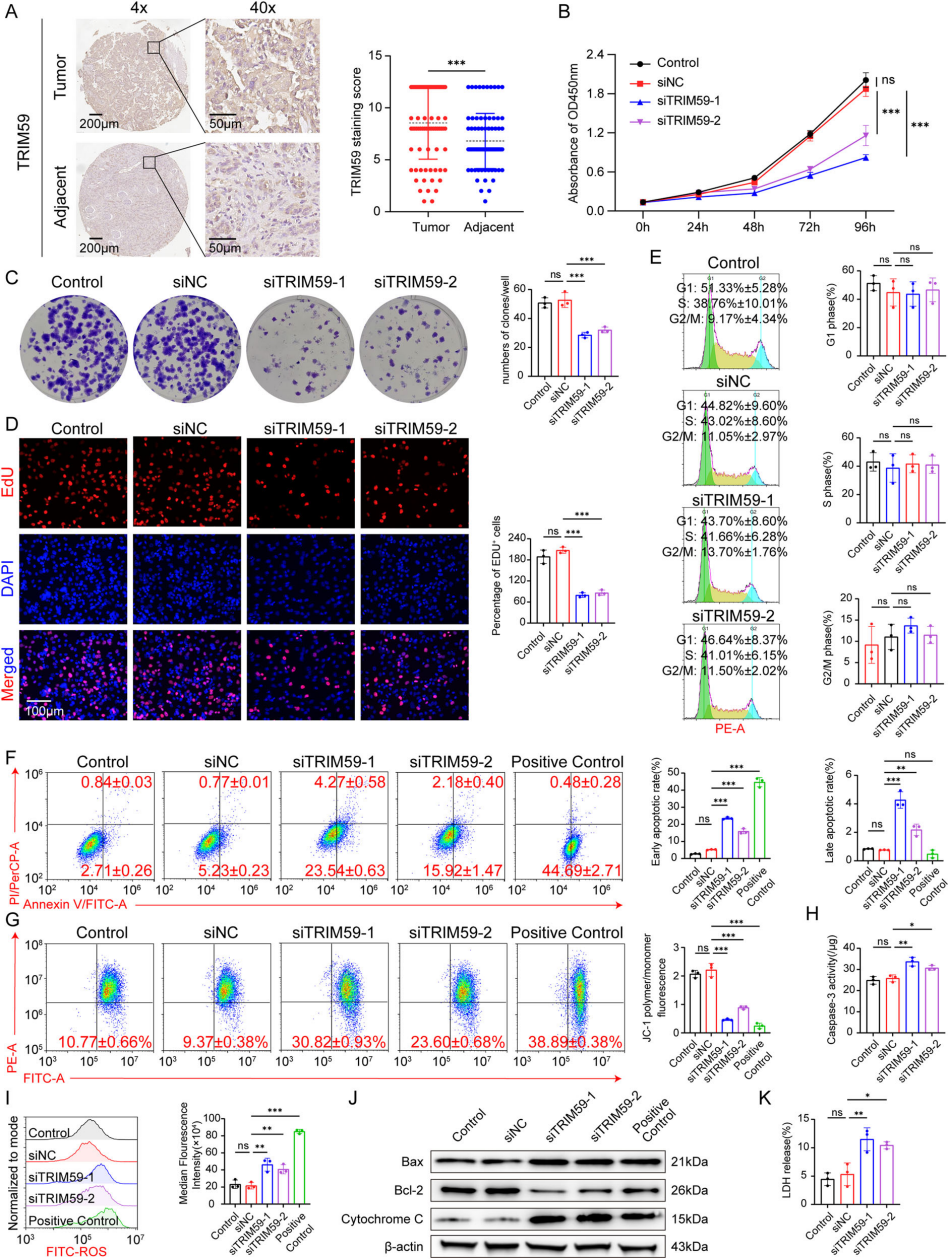
TRIM59 suppresses the mitochondria related apoptosis of papillary renal cell carcinoma in ACHN cells. **A** Representative images of TRIM59 immunohistochemistry (IHC) staining in papillary renal cell carcinoma (pRCC) tissue microarray (including 80 human pRCC cancer tissues and adjacent paired normal tissues), and the quantitative analysis of TRIM59 protein levels. Scale bar, 50 μm. **B**–**D** TRIM59 silencing contributed to attenuating proliferation of ACHN cells, which was assayed by CCK-8 assay (**B**), colony formation assay (**C**) and EdU (**D**) staining. **E** Cell-cycle phase proportions were detected by flow cytometry after knocking TRIM59 in ACHN cells, comparing to negative control. **F** Cell apoptosis followed by TRIM59 silencing was assayed via Annexin V/PI double staining in ACHN cells, and the quantitative analysis of the ratio of the apoptotic cells. (Positive Control: carboplatin 80 μM for 48 h). **G** TRIM59 silencing reduced the mitochondrial membrane potential (MMP) in ACHN cells. MMP was measured by flow cytometry using JC-1 staining. Red fluorescence (PE channel) represented polymers and green fluorescence (FITC channel) represented monomers. (Positive Control: CCCP 50 μM for 15 minutes). **H** Caspase-3 activity assay was utilized to determine apoptosis after knocking-down TRIM59 in ACHN cells. **I** Flow cytometry (left) and statistical analysis (right) show the ROS levels after TRIM59 knockdown in ACHN cells. (Positive Control: Rosup 100 μM for 4 h). **J** Western blot detection of expression levels of Bax, Bcl-2, Cytochrome C, and β- actin after TRIM59 knockdown in ACHN cells. (Positive Control: carboplatin 80 μM for 48 h). **K** LDH assay was utilized to determine apoptosis after knocking-down TRIM59 in ACHN cells. The experiments were repeated 3 times independently. Data are presented as the mean ± s.d.; *P < 0.05; **P < 0.01; ***P < 0.001.

Immunohistochemical staining of TRIM59 was conducted on a tissue chip of pRCC, including 80 samples of pRCC and adjacent tissue. Results showed that TRIM59 was highly expressed in pRCC tissues compared to adjacent normal tissues (*P* < 0.001), suggesting that TRIM59 may function as an oncogene in pRCC (Fig. 4A). TRIM59 was also highly expressed in the pRCC cell line ACHN compared to the normal renal epithelial cell line HEK-293T (Figure S4A). siRNAs were used to knock down TRIM59 expression in both ACHN and Caki-2 cell lines (Figure S4B–E). Cell proliferation assays revealed that TRIM59 knockdown suppressed the proliferation of ACHN cells (Fig. 4B) and Caki-2 cells (Figure S4F). Additionally, colony formation ability was inhibited upon TRIM59 silencing (Fig. 4C, Figure S4G). The number of EdU-labeled cells in TRIM59 siRNA groups significantly decreased compared to controls (Fig. 4D, Figure S4H), indicating reduced DNA synthesis and inhibited proliferation following siTRIM59 treatment. We then examined the relationship between TRIM59 and the cell cycle, which was strongly linked to RiskScore (Fig. 4E). Surprisingly, analysis of cell cycle phases showed that TRIM59 knockdown did not significantly alter the G1, S, or G2/M phases, suggesting that TRIM59 was not involved in cell cycle arrest in pRCC.

Notably, apoptosis rates in the siTRIM59 groups were substantially higher than in the Control group (Fig. 4F, Figure S4I). To determine the stage of apoptosis following TRIM59 silencing, we used JC-1 reagent to assess mitochondrial membrane potential (MMP) changes. The MMP reduction in siTRIM59 groups was significantly greater than in the Control group (Fig. 4G, Figure S4J), indicating early apoptosis. Apoptosis is primarily mediated by three classical pathways: mitochondrial, endoplasmic reticulum, and death receptor pathways, each triggered by distinct initiating factors [24]. To further elucidate the mechanism of TRIM59-induced apoptosis, we found that siTRIM59 treatment led to increased levels of caspase-3 activity (Fig. 4H, Figure S4K), ROS levels (Fig. 4I, Figure S4L), Bax, and cytochrome c protein, accompanied by a marked reduction in Bcl-2 expression (Fig. 4J, Figure S4M), suggesting that apoptosis may occur via the mitochondrial pathway. In addition, elevated LDH activity in the siTRIM59 group indicated increased cytotoxicity (Fig. 4K, Figure S4N).

### TRIM59 mediates the ubiquitination and degradation of ACAT1 in papillary renal cell carcinoma cells

**Fig. 5:**
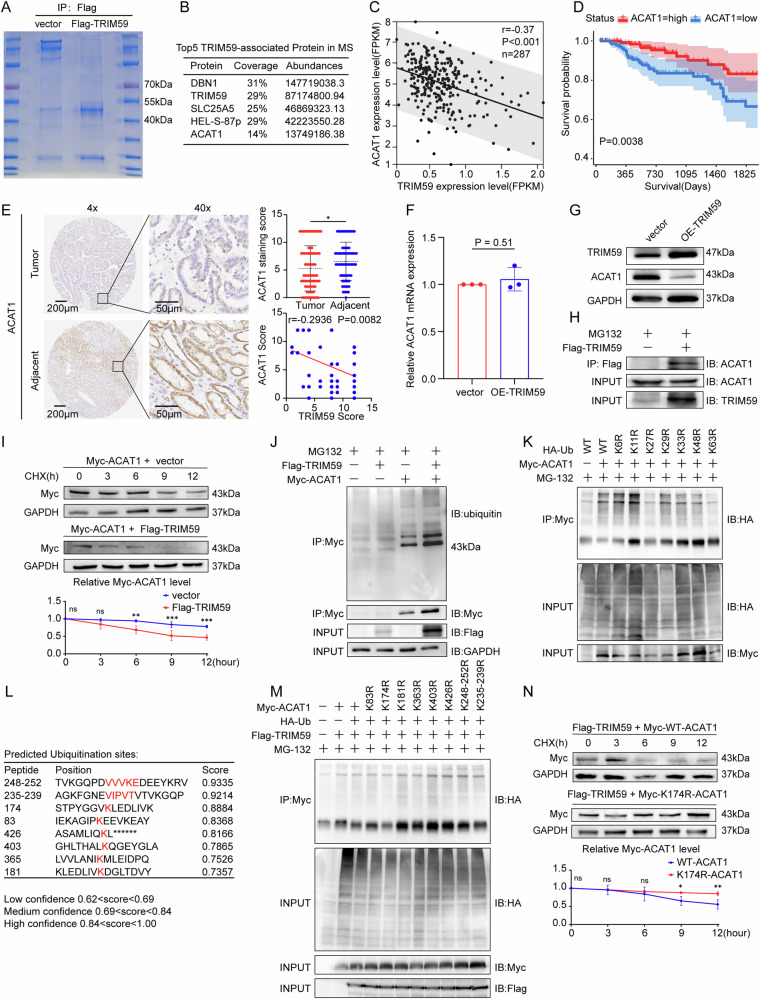
TRIM59 mediates the ubiquitination and degradation of ACAT1 in papillary renal cell carcinoma cells. **A** HEK-293T cells were transfected with Flag-TRIM59 or an empty vector for 48 h harvested (before collection treated with MG132 20 μM for 6 h, and the lysates were subjected to anti-Flag immunoprecipitation (IP) and analyzed by Coomassie Brilliant Blue for mass spectrometry assay. **B** TOP5 TRIM59-interacted proteins were identified by MS analysis. **C** Correlation between TRIM59 and ACAT1 in pRCC patients (*n* = 287) from TCGA database. **D** Kaplan–Meier survival curves of the 5-year overall survival (OS) rate based on patients with high (ACAT1 = high) or low (ACAT1 = low) expression of ACAT1 in pRCC. **E** Representative images of ACAT1 IHC staining in pRCC tissue microarray (including 80 human pRCC cancer tissues and adjacent paired normal tissues), The quantitative analysis of ACAT1 protein levels and the negative correlation between TRIM59 and ACAT1 were also presented. Scale bar, 50 μm. **F**, **G** TRIM59 reduced ACAT1 protein level without affecting mRNA expression of ACAT1. The mRNA (**F**) and protein (**G**) levels of ACAT1 in wild-type (WT) and overexpressed TRIM59 ACHN cells were measured by qRT-PCR and western blot analysis, respectively. **H** HEK-293T cells were transfected with exogenous Flag-TRIM59 and Myc-ACAT1. Anti-Flag and anti-Myc antibodies were used for immunoprecipitation to detect the mutual effect between TRIM59 and ACAT1. **I** Western blot analysis demonstrated ACAT1 protein decay at the indicated time points after cycloheximide (50 μg/ml) addition to vector or overexpressed TRIM59 ACHN cells. The line chart was further quantitatively analyzed myc-ACAT1 expression. **J** IP and western blot showed the effect of TRIM59 overexpression on the ubiquitination of ACAT1 in HEK-293T cells. **K** HA-WT, K6, K11, K27, K29, K33, K48 or K63 Ub were co-transfected with Myc-ACAT1 and Flag-TRIM59 into HEK293T cells. After treatment with 20 µm MG132 for 6 h, cell lysates were subjected to ubiquitination assay, and the ubiquitination level of ACAT1 detected by HA antibody. **L** The ubiquitination sites of ACAT1 were predicted by BDM-PUB and UBPRED database. **M** Immunoblotting was performed to detect the ubiquitination of ACAT1 mutants in HEK293T cells that were co-transfected with Myc-ACAT1 mutants, Flag-TRIM59, and HA-Ub. **N** The K174R mutation prevents TRIM59 from mediating ACAT1 degradation. The line chart was further quantitatively analyzed myc-ACAT1 expression. The experiments were repeated 3 times independently. Data are presented as the mean ± s.d.; *P < 0.05; **P < 0.01; ***P < 0.001.

Previous studies have shown that TRIM59 has E3 ubiquitin ligase activity. To identify substrate proteins ubiquitinated by TRIM59, we performed an IP assay followed by mass spectrometry to profile TRIM59-interacting proteins (Fig. 5A). Among the identified interactions, DBN1, TRIM59, SLC25A5, HEL-S-87p, and ACAT1 were the top five associated proteins (Fig. 5B). Among these, SLC25A5, HEL-S-87p, and ACAT1 function as metabolite transporters or key enzymes involved in metabolic regulation [25–29], while DBN1 is closely related to muscle contraction and neuron migration [30, 31]. Given the strong link between renal cancer and metabolism, we prioritized SLC25A5, HEL-S-87p and ACAT1 as our metabolic targets. Unexpectedly, our study confirmed that TRIM59 did not affect SLC25A5 or HEL-S-87p expression (Figure S5A) but significantly reduced ACAT1 expression, leading us to focus subsequent investigations on ACAT1 as a downstream target of TRIM59. In the KIRP dataset from TCGA, a negative correlation was found between TRIM59 and ACAT1 gene expression levels (Spearman r = -0.37, *P* < 0.001) (Fig. 5C), and low ACAT1 expression was significantly associated with poor OS in KIRP (*P* = 0.0038) (Fig. 5D).

IHC staining of tissue chips demonstrated a significant downregulation of ACAT1 protein levels in pRCC tissues compared to adjacent normal tissues, with TRIM59 expression negatively correlated with ACAT1 in pRCC tissues (Fig. 5E). To investigate the mechanism underlying ACAT1 downregulation in pRCC, empty vector and Flag-TRIM59 plasmids were overexpressed in ACHN cells, followed by analysis of ACAT1 mRNA and protein levels. Notably, the mRNA expression of ACAT1 remained consistent with the control group (*P* = 0.51) (Fig. 5F), whereas ACAT1 protein levels were decreased in the Flag-TRIM59 group (Fig. 5G). These findings led us to propose that TRIM59 might promote ACAT1 protein degradation via ubiquitination.

To test this hypothesis, co-immunoprecipitation (Co-IP) confirmed an interaction between TRIM59 and ACAT1 (Fig. 5H). The turnover rate of ACAT1 was assessed with or without TRIM59 by a cycloheximide (CHX) chase assay to determine the impact of TRIM59 on ACAT1 protein stability. Overexpression of TRIM59 in ACHN cells significantly reduced the half-life of exogenous ACAT1 protein (Fig. 5I). Further Co-IP analysis revealed that co-expression of TRIM59 and ACAT1 led to a significant increase in ACAT1 ubiquitination (Fig. 5J).

Ubiquitin contains seven lysine residues, enabling site-specific ubiquitination at K6, K11, K27, K29, K33, K48, or K63. Co-transfection with Ub point mutation plasmids and ACAT1 significantly enhanced the ubiquitination signal of Myc-ACAT1, except for mutations K27R and K63R (Fig. 5K). These findings suggested that ACAT1 was ubiquitinated through K27 and K63. Additionally, bioinformatic analysis was used to predict potential ubiquitination sites on ACAT1 (http://sumosp.biocuckoo.org/index.ph) (Fig. 5L). Eight lysine residues (K83, K174, K181, K363, K403, K426, K248-K252, and K235-K239) were predicted as potential ubiquitination sites on ACAT1, and mutant vectors for each site were generated for validation. Mutation at the K174 residue notably reduced ACAT1 ubiquitination (Fig. 5M), indicating that K174 is a critical residue for K27- and K63-linked ubiquitination of ACAT1 facilitated by TRIM59. Notably, after mutating the K174 site, TRIM59 was unable to alter ACAT1 protein stability.

### TRIM59 inhibits mitochondria related apoptosis through ACAT1-cardiolipin metabolism pathway

**Fig. 6:**
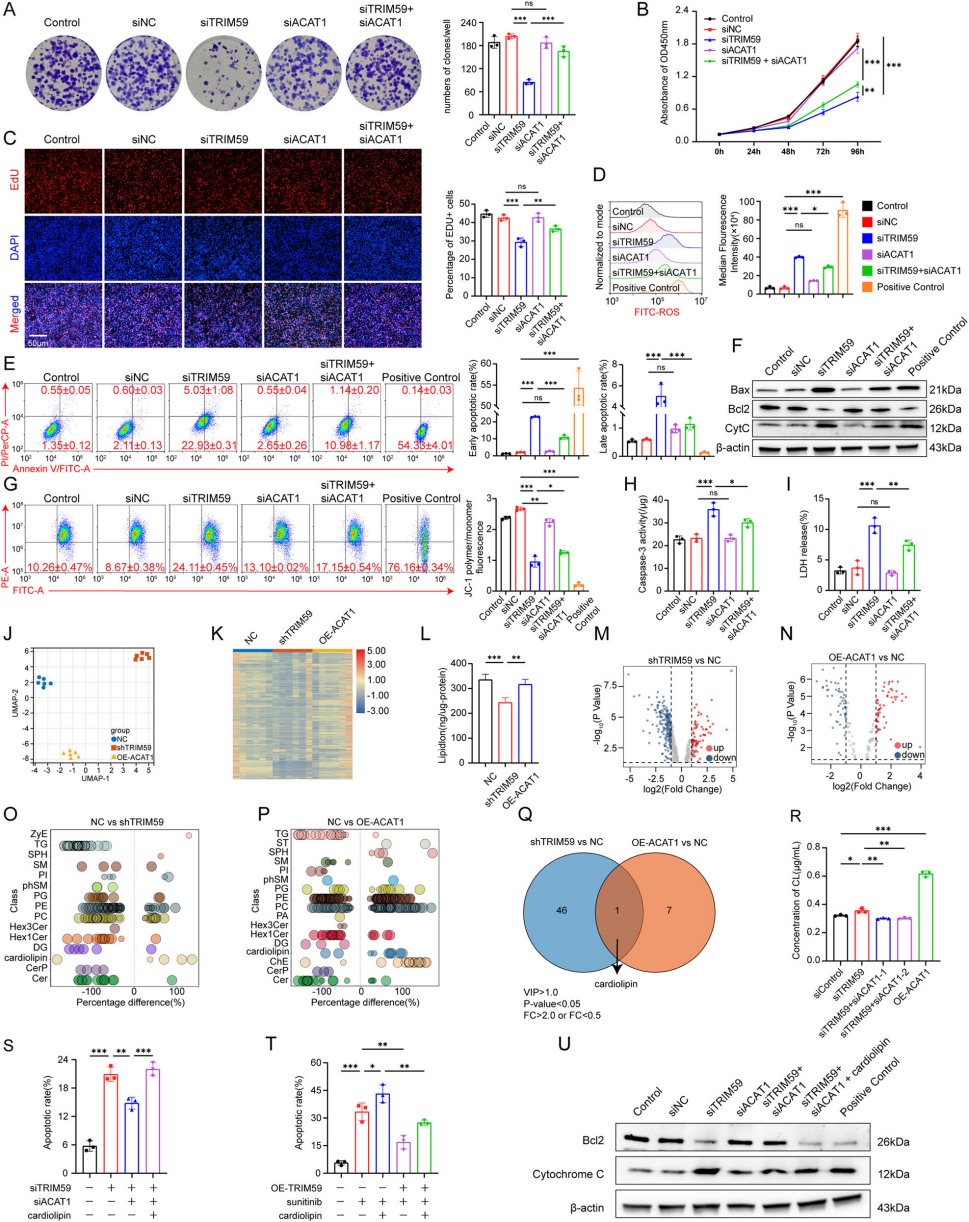
TRIM59 inhibits mitochondria related apoptosis through ACAT1-cardiolipin metabolism pathway. **A**–**D** TRIM59 silencing exerted the cell proliferation via ACAT1, which was evaluated by colony formation assay (**A**), CCK-8 assay (**B**), EdU staining (**C**) and ROS levels (**D**) to detect the proliferation of ACHN cells, and the quantitative analysis of colony formation assay and EdU staining were also presented. **E** Cell apoptosis after TRIM59 silencing with or without knocking-down ACAT1 meanwhile via Annexin V/PI double staining in ACHN cells. (Positive Control: carboplatin 80 μM for 48 h). **F** Western blot detection of the expression levels of Bax, Bcl-2, Cytochrome C, and β- actin in ACHN cells after silencing TRIM59 with or without ACAT1 knockout. (Positive Control: carboplatin 80 μM for 48 h). **G** The change of mitochondrial membrane potential followed by TRIM59 silencing with or without knocking-down ACAT1 meanwhile was detected by flow cytometer using JC-1 staining. (Positive Control: CCCP 50 μM for 15 minutes). **H**, **I** Apoptosis was determined by Caspase-3 activity assay (**H**) and LDH assay (**I**) in ACHN cells. **J** PCA plot showed clustering of substantial disparities in lipid metabolism across negative control group, shTRIM59 group and over-expression ACAT1 group. **K** The hierarchical clustering results of different lipids in the NC group, shTRIM59 group, and OE ACAT1 group. **L** The overall lipid content level in the NC group, shTRIM59 group, and OE-ACAT1 group, which indicated that the total lipid content in the shTRIM59 group was less than in the control and OE-ACAT1 group. **M**, **N** Volcano plots showed the differential intensity of lipid metabolites between shTRIM59 group and NC group (**K**), as well as OE-ACAT1 group and NC group (**L**). **O**, **P** Bubble charts illustrated the variations in lipid classes between NC group and shTRIM59 group (**M**), as well as NC group and OE-ACAT1 group (**N**). **Q** The Venn diagram illustrated the common differential lipid metabolite cardiolipin, which was enriched in the differential lipid subclasses of the shTRIM59 group relative to the NC group, as well as in the OE-ACAT1 group compared to the NC group. **R** The ELISA assay of cardiolipin content confirmed that TRIM59 inhibited cardiolipin synthesis in ACHN cells by regulating the level of ACAT1. **S** Rescue experiment revealed the effect of TRIM59 silencing on cell apoptosis in ACHN cells could be rescued through knocking-down ACAT1 or supplement of cardiolipin (50 μM, 6 h). **T** Overexpressed TRIM59 reduced cell apoptosis induced by sunitinib, but was partly counteracted by supplement of cardiolipin (50 μM, 6 h) in ACHN cells. **U** Western blot detection of the expression levels of Bcl-2, Cytochrome C, and β- actin after rescue experiments in ACHN cells. (Positive Control: carboplatin 80 μM for 48 h). The experiments were repeated 3 times independently. Data are presented as the mean ± s.d. *P < 0.05; **P < 0.01; ***P < 0.001.

To verify that pRCC tumor development was regulated by the interaction between TRIM59 and ACAT1, rescue experiments were performed on ACHN cells after simultaneous silencing of TRIM59 and ACAT1. Cloning formation (Fig. 6A), EdU assay (Fig. 6B), CCK8 (Fig. 6C) and ROS levels (Fig. 6D) demonstrated that the inhibition of cell proliferation due to TRIM59 knockdown was partially reversed by knocking down ACAT1. Furthermore, simultaneous silencing of both TRIM59 and ACAT1 reduced the apoptosis rate in ACHN cells compared to the TRIM59-silenced group (Fig. 6E). Additionally, These findings were further supported by changes in the expression of Bax, Bcl-2, and cytochrome c (Fig. 6F), partial recovery of mitochondrial membrane potential (Fig. 6G), and corresponding alterations in caspase-3 activity(Fig. 6H) and LDH release (Fig. 6I).

ACAT1 is a mitochondrially localized enzyme that catalyzes the reversible formation of acetoacetyl-CoA from two molecules of acetyl-CoA, which plays crucial roles in isoleucine catabolism, ketogenesis, ketolysis, and fatty acid oxidation. A lipidomics study was conducted in ACHN cells with stable TRIM59 knockdown and ACAT1 overexpression to clarify the biological link between TRIM59 and lipid metabolism. Principal component analysis (PCA) revealed substantial disparities in lipid metabolism across the TRIM59 knockdown, ACAT1 overexpression, and control groups (Fig. 6J). A systematic classification was performed to examine lipid content thoroughly in these groups, enabling precise identification of marker lipids and analysis of alterations in associated metabolic processes (Figure S6A–C). Results indicated that total lipid content in the shTRIM59 group was lower than in the control and OE-ACAT1 groups (Fig. 6K, L). Volcano plots clearly illustrated the differential intensity of lipid metabolites between the two groups (Fig. 6M, N). Among the differential lipid metabolites, concentrations of triglycerides (TG), sphingomyelins (SM), phosphatidylglycerol (PG), phosphatidylethanolamine (PE), phosphatidylcholine (PC), ceramides (Cer), and diglyceride (DG) were significantly reduced in the shTRIM59 group compared to the control group, whereas cardiolipin was elevated (Fig. 6O). However, in the OE-ACAT1 group, the concentrations of sterol (ST), sphingolipid (SPH), PG, PC, cardiolipin, and cholesterol ester (ChE) exceeded those in the control group, while TG and Cer were reduced (Fig. 6P). A Venn diagram analysis was performed for NC vs. OE-ACAT1 and NC vs. shTRIM59 groups (VIP > 1.0, *P* < 0.05, FoldChange > 2.0 or FoldChange < 0.5) (Fig. 6Q). Cardiolipin was the only lipid metabolite that varied across all three groups (Figure S6D). To validate this outcome, the supernatant and precipitate from each cell group were collected for ELISA to quantify cardiolipin content. Results indicated that cardiolipin levels in the siTRIM59 and ACAT1 overexpression groups were elevated relative to the control group but decreased following ACAT1 knockdown in the siTRIM59 group (Fig. 6R). These findings further validated TRIM59’s ability to suppress cardiolipin synthesis in ACHN cells by regulating ACAT1 levels.

More importantly, cardiolipin has been identified as a key signal for initiating apoptosis [32] and mitochondrial autophagy [33], being exposed on the mitochondrial outer membrane in response to various mitochondrial stresses [34, 35]. Interference with cardiolipin can induce dissociation of the complex between cytochrome c and cardiolipin, leading to subsequent release of cytochrome c into the cytoplasm, which triggers downstream cascades associated with apoptosis [36–38]. Therefore, based on interference with TRIM59 and ACAT1, we further added cardiolipin to assess apoptosis changes in ACHN cells. Results confirmed that cardiolipin could restore apoptosis levels diminished by TRIM59 overexpression and those reduced by ACAT1 knockdown following TRIM59 knockdown (Fig. 6S, T, Figure S6D, E). Moreover, cardiolipin supplementation led to decreased expression of the anti-apoptotic gene Bcl-2 and increased expression of cytochrome c (Fig. 6U), further supporting its role in reactivating mitochondria-mediated apoptosis.

### Knockdown TRIM59 inhibits pRCC tumorigenesis by ACAT1-cardiolipin pathway in vivo

**Fig. 7:**
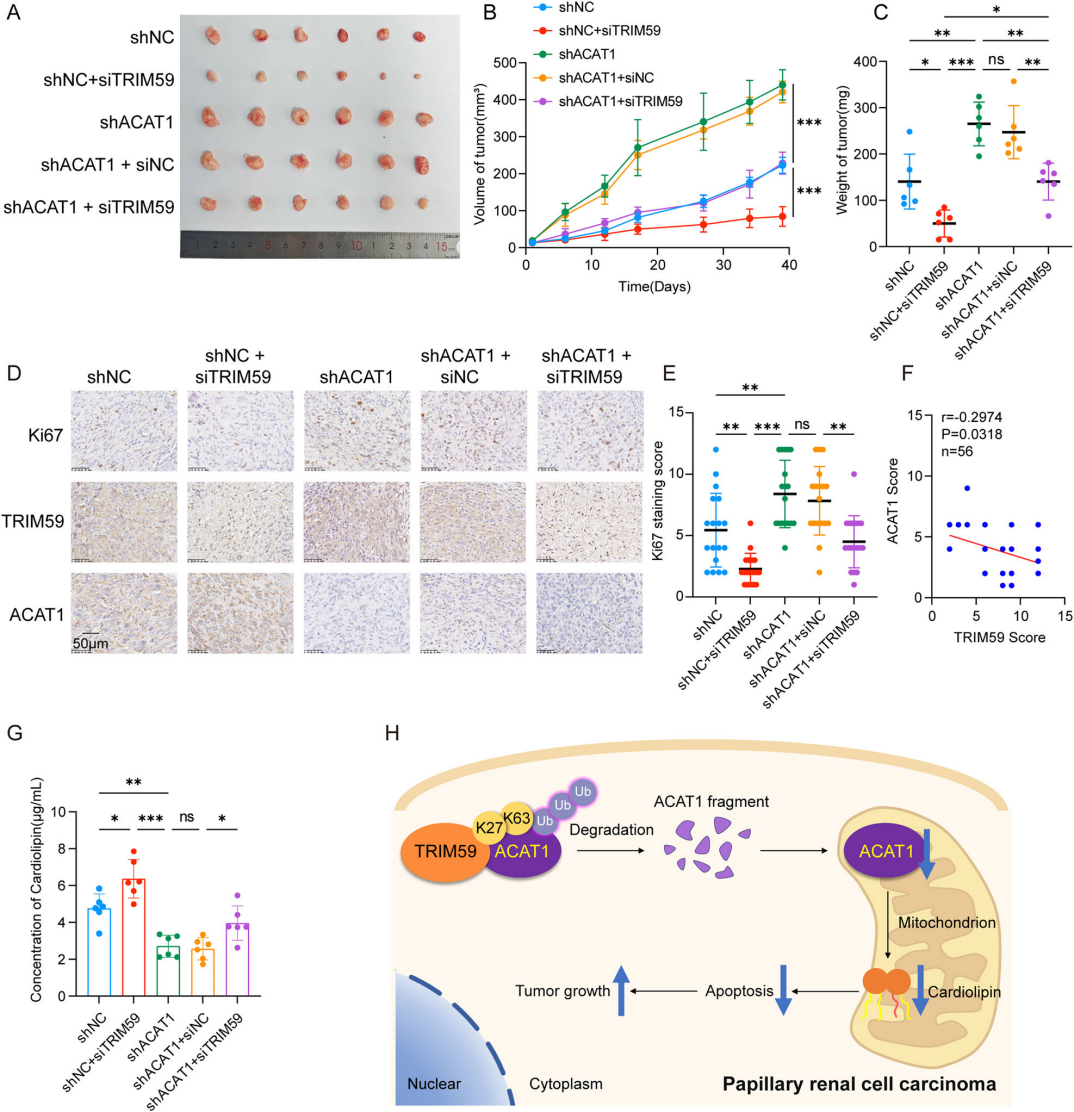
Knockdown TRIM59 inhibits pRCC growth by ACAT1-cardiolipin pathway in vivo. **A**–**C** Knockdown of ACAT1 increased tumor growth, while siTRIM59 peritumoral injection (5 nmol per injection, three times per week) further reduced the tumor burden in nude mice. The harvested tumors were shown in (**A**). Growth curves (**B**) and tumor weights (**C**) were mean ± s.d. of seven mice in each group. **D** IHC staining detected the expression of Ki67, TRIM59 and ACAT1 in ACHN xenograft tissues. Scale bars, 50 μm. **E** The quantitative analysis of Ki67 protein levels was presented. **F** The negative correlation between TRIM59 and ACAT1 was presented. **G** The ELISA assay of cardiolipin content in tumor tissue supernatants. **H** Graphical illustration of the working model. Data are presented as the mean ± s.d.; *P < 0.05; **P < 0.01; ***P < 0.001.

To further investigate the roles of TRIM59 and ACAT1 in tumorigenesis, xenograft experiments were conducted. Stable ACAT1 knockdown and negative control constructs were created in ACHN cells, which were subsequently transplanted subcutaneously into nude mice. Once tumors became palpable, optimized siTRIM59 and control treatments were injected in and around the tumors three times per week (5 nmol per injection). Four weeks after cell injection, tumor growth was significantly increased in mice with stable ACAT1 knockdown, whereas TRIM59 knockdown—either alone (shNC+siTRIM59) or combined with ACAT1 knockdown (shACAT1+siTRIM59)—led to a marked reduction in both tumor volume and weight compared to their respective controls (Fig. 7A–C). These findings highlight the tumor-suppressive effect of TRIM59 silencing in vivo.

Immunohistochemistry staining further showed increased expression of the proliferation marker Ki67 in tumors from ACAT1 knockdown mice, which was reduced when TRIM59 was simultaneously silenced in ACAT1 knockdown mice. A reduction in Ki67 expression was also observed in the shNC+siTRIM59 group compared to negative control (Fig. 7D, E). Additionally, the negative correlation between TRIM59 and ACAT1 expression was statistically confirmed (Fig. 7F). Moreover, compared to controls, cardiolipin expression was significantly reduced in ACAT1 knockdown tumors but increased in siTRIM59-injected tumors (Fig. 7G). These findings support the observed positive correlation between ACAT1 expression and prognosis in pRCC patients, while TRIM59 expression was negatively associated with patient prognosis.

## Discussion

From both pathological and molecular genetic perspectives, pRCC is recognized as a distinct subtype within RCC and is often asymptomatic or presents with non-specific symptoms in its early stages. By the time patients experience symptoms such as abdominal pain, hematuria, or abdominal masses, the disease has frequently advanced, leading to poor prognosis and high mortality. However, effective targeted therapies for pRCC remain elusive due to limited understanding of its specific molecular pathogenesis. Evidence suggests that abnormal gene expression and mutations are associated with pRCC development. For instance, pRCC often exhibits recurrent alterations in MET due to gene amplification or mutations. Abnormal MET activation can lead to tumorigenesis, metastasis, and increased invasiveness [39]. Due to MET overexpression in pRCC, some MET kinase inhibitors, such as Savolitinib and Crizotinib, have been used in metastatic pRCC patients. However, compared to the current first-line treatment with Sunitinib, neither Savolitinib nor Crizotinib improved progression-free survival (PFS) in metastatic pRCC [40]. It is worth noting that these medications have a high probability of grade 3 or 4 adverse events, which often affect patient adherence to treatment [41].

Identifying predictive markers for pRCC and providing tailored therapeutic strategies are therefore essential to improving patient prognosis. This study is the first to establish a prognostic RiskScore model based on TRIM family gene expression in pRCC, offering a more accurate tool for stratifying patient outcomes and monitoring recurrence. Additionally, it provides insights to aid clinical decision-making for potentially high-risk patients. Within this model, TRIM59 emerged as a pivotal risk gene significantly associated with poorer prognosis. Compared to other TRIM family members in the model, TRIM59 demonstrated a stronger correlation with aggressive clinical features and advanced stages of pRCC. Thus, we hypothesized that TRIM59 plays a significant regulatory role in pRCC and may serve as a specific target for diagnosis and therapy.

Previous studies have demonstrated the oncogenic role of TRIM59 in various malignancies [42, 43]. Abnormal lipid metabolism significantly contributes to renal cancer progression; however, the relationship between TRIM59 and lipid metabolism has not been reported. Our study showed that TRIM59 knockdown in pRCC regulates lipid metabolism by mediating the ubiquitination of ACAT1 at K174, thereby promoting mitochondrial-associated apoptosis. ACAT1, a critical enzyme in fatty acid oxidation. Previous research has shown that ACAT1 has a dual role in tumorigenesis. ACAT1 inhibitors effectively suppress tumor proliferation with elevated ACAT1 expression; conversely, diminished ACAT1 expression in the tumor microenvironment leads to MDSC accumulation, creating an immunosuppressive environment that facilitates glioblastoma progression [44]. Furthermore, ACAT1 was found to be downregulated in ccRCC [29], consistent with our findings in pRCC. However, the mechanism of ACAT1 and ACAT1-mediated lipid metabolism in pRCC remains unclear.

Through lipidomics analysis, we identified cardiolipin as a potential ACAT1 target. Cardiolipin is a mitochondria-specific phospholipid crucial in mitochondria-dependent apoptosis. Some studies have shown that tetralinoleoyl cardiolipin (TLCL), one of the most abundant cardiolipins in liver cancer tissues, is significantly reduced [38]. Furthermore, TLCL oxidation produces active lipid electrophiles, such as 4-hydroxy-nonenal (4-HNE), which influence apoptosis. Hepatocellular carcinoma cell lines treated with TLCL exhibited a significant increase in apoptosis susceptibility. Notably, cardiolipin is associated with apoptosis induction, and apoptosis activation can modify cardiolipin distribution and concentration [45]. In the early stages of apoptosis, cardiolipin translocates from the inner to the outer mitochondrial membrane, promoting cytochrome c binding and increasing its peroxidase activity, thereby propelling the apoptotic process [46]. Additionally, apoptosis initiation accelerates cardiolipin’s metabolic cycle, reducing its mitochondrial levels and promoting its breakdown and recycling [47].

Our research demonstrated that TRIM59 facilitates ACAT1 ubiquitination in pRCC, thereby reducing endocytic phospholipid synthesis and inhibiting mitochondria-dependent apoptosis, ultimately promoting tumor growth (Fig. 7H). However, we did not investigate how ACAT1 affects cardiolipin content or the specific mechanism by which cardiolipin subtypes facilitate mitochondria-dependent apoptosis. A substantial number of clinical cases and further refinement of risk models are still needed to assess clinical relevance. Further laboratory studies are also essential to elucidate the molecular mechanisms of ACAT1 and cardiolipin in mitochondrial metabolism and tumorigenesis.

Our findings also provide several potential avenues for therapeutic innovation in pRCC. Given that TRIM59 functions as an E3 ubiquitin ligase and is significantly associated with poor prognosis, it may serve as a viable molecular target. Targeting TRIM59—through small molecule inhibitors, RNA interference, or emerging approaches such as PROTACs—could potentially restore apoptosis sensitivity in tumor cells. Moreover, ACAT1, a key enzyme in lipid metabolism, has previously been investigated in other cancer types as a druggable target. Our results suggest that regulating the TRIM59–ACAT1–cardiolipin axis may offer a novel strategy to modulate mitochondrial-mediated apoptosis in pRCC.

## Material and methods

### Kaplan-Meier Plotter survival analysis

High-throughput RNA-seq data of pRCC patients and normal renal tissue samples were obtained from The Cancer Genome Atlas (TCGA) via the University of California, Santa Cruz Genome Browser (UCSC) Xena. TRIM family gene expression levels in pRCC and normal renal tissues were analyzed, with age, type, stage, gender, grade, recurrence, tumor status, and gene expression relationships visualized in a heat map. The association between TRIM gene family expression and overall survival (OS) or progression-free survival (PFS) of pRCC patients was examined using the KM-plotter database, and a forest plot was generated with the forestplot R package. The survival curve was based on the relationship between TRIM expression and OS in the TCGA database.

### Construction of RiskScore model

Using TRIM family gene expression profiles and OS survival data, a multi-gene risk model was developed via LASSO Cox analysis to predict pRCC patient prognosis. Univariate and multivariate Cox regression analyses evaluated the relationship between the RiskScore model, clinical variables (age, stage, recurrence, gender), and OS.

### Construction and verification of nomogram

The nomogram provided a more intuitive and practical visualization of the risk model, aiding clinical outcome prediction. Constructed from significant multivariate Cox regression clinical features, including RiskScore, recurrence, and stage, the nomogram’s prognostic classification accuracy for 3-year and 5-year survival was assessed using the timeROC R package, with ROC curves displayed.

### GO and KEGG pathway analyses

GO and KEGG enrichment pathway analyses were conducted on the cross-genes using the DAVID database (https://david.ncifcrf.gov/). The official gene symbol was used as the identifier, and *Homo sapiens* was selected as the species. Pathway enrichment was screened based on count values and a significance threshold of *P* < 0.05.

### Gene set enrichment analysis (GSEA)

Papillary renal cell carcinoma samples from TCGA and normal renal tissue samples from GTEx were obtained from UCSC Xena. Differential expression analysis was performed using the limma R package, and the clusterProfiler R package was employed for GSEA.

### PPI interaction network construction

Protein-protein interactions of TRIM family genes were analyzed using the STRING database (https://string-db.org/).

### Cell culture and treatment

The pRCC cell lines were sourced from Meisen Chinese Tissue Culture Collections. ACHN, HEK-293T, HK-2, Caki-1, Caki-2 and 786-O cells were obtained from the American Type Culture Collection (ATCC, Manassas, VA, USA). ACHN, HEK-293T, HK-2, Caki-1 and 786-O cells were cultured in DMEM (Gibco, USA) supplemented with 10% fetal bovine serum (FBS) and incubated at 37 °C in a humidified atmosphere with 5% CO₂. Caki-2 cells were cultured in McCoy’s 5 A medium (Gibco, USA) with 10% FBS at 37 °C in 5% CO₂. Culture dishes were purchased from Corning (New York State, USA). All cell lines were routinely screened for mycoplasma contamination using the PCR Mycoplasma Detection kit (40612ES60, YEASEN, China). Only early passage cells were used in experiments.

### Western blot analysis

Cells were lysed in RIPA buffer containing a protease inhibitor (05892791001, Roche, Basel, Switzerland) and incubated in 1.5 mL EP tubes at 4 °C for 15 min. Protein concentrations in the lysates were measured with a BCA protein assay kit (A55860, Thermo Scientific, Massachusetts, USA) and normalized to equal amounts of protein. Cell lysates were separated by 10% SDS-PAGE, transferred to 0.2 μm PVDF membranes (03010040001, Roche, Switzerland), and probed with the specified primary antibodies. After probing, the blots were incubated with species-specific secondary antibodies and visualized using ECL (A38555, Thermo Scientific, USA).

### Antibodies

Anti-TRIM59 (28575-1-AP), anti-Flag-Tag (20543-1-AP), anti-GAPDH (60004-1-Ig), anti-beta Actin(66009-1-Ig), anti-rabbit (SA00001-2) and anti-mouse (SA00001-1) secondary antibodies, were purchased from Proteintech (Wuhan, China). Anti-ACAT1 (ab312320) was obtained from Abcam. Anti-Flag-Tag (F-3165) was sourced from Sigma (Cambridge, UK). Anti-Myc-Tag (MA1-21316), anti-HA-Tag (26183), and anti-IgG (MA5-42729) were purchased from Thermo Fisher Scientific (USA). Anti-bax(F0037), anti-Bcl-2(F0125), and anti-Cytochrome C(F0194) were purchased from Selleck(Texas, USA).

### Plasmids and siRNA

SiNC (UUCUCCGAACGUGUCACGU), siTRIM59-1 (UCUCCAAAAAGGAUGUCAU), siTRIM59-2 (CAACUGGCAUUGAAUCUUU), siACAT1-1 (GCUGAAUAUUGCACGAAAU), siACAT1-2 (GCUGCAUCUAUGGUUCUUA), pLKO.1-puro-shTRIM59 (GGAAGCTGTTCTCCAGTAT), were purchased from IGEbio (Guangzhou, China). pReceiver-Lv242-Flag-TRIM59 was purchased from igenebio (Guangzhou, China). pcDNA3.1-Myc-ACAT1 (wild-type, K83R, K174R, K181R, K365R, K403R, K426R, VVVKE-248-252-RRRRR, VIPVT-235-239-RRRRR mutant), pcDNA3.1-HA-Ub (wild-type, K6R, K11R, K27R, K29R, K33R, K48R, K63R mutant) were purchased from Ruibo (Guangzhou, China). pLKO.1-tet on-shACAT1 were purchased from Tsingke (Guangzhou, China).

### RNA extract, reverse transcription‑PCR, and qRT‑PCR

Total RNA was extracted from cultured cells using the RNA Quick Purification Kit (ESScience, Shanghai, China) and reverse-transcribed into complementary DNA (cDNA) using a reverse transcription reagent (Vazyme, Nanjing, China) according to the manufacturer’s instructions. RT-PCR was performed on the Bio-Rad T100 system. qRT-PCR was conducted using ChamQ SYBR Color qPCR Master Mix (Vazyme, China). cDNA served as the template for amplification with specific primers. qRT-PCR was performed using the Thermo Scientific QuantStudio detection system. The fold change of genes was calculated using the 2^-ΔΔCt^ method. GAPDH was used as a loading control. The primer used are: TRIM59, sense: 5´TCCTCGTGTACTGCCAT-3´, antisense: 5´-CAATGCCAGTTGGAGCAATTTC-3´. ACAT1, sense: 5´-CCAGAAGTAAAGCAGCATGGG-3´, antisense: 5´-ATCTGCCGTCATGAGAACCA-3´. GAPDH, sense: 5´-TGCACCACCAACTGCTTA-3´, antisense: 5´-GGATGCAGGGATGATGTTC-3´.

### Cell cycle assay

The cell cycle assay was performed using a cell cycle assay kit (E-CK-A351, Elabscience, Wuhan, China) according to the manufacturer’s protocol. DNA content was analyzed with NovoCyte flow cytometry (Agilent, California, USA), and the percentage of cells in each cell cycle phase was determined using NovoExpress software (Agilent, USA).

### Immunohistochemistry (IHC) staining

IHC staining was conducted using TRIM59 (28575-1-AP, Proteintech, China) and ACAT1 (ab312320, Abcam, UK) antibodies following standard protocols on formalin-fixed and paraffin-embedded tumor tissues. TRIM59 antibody was used at a 1:500 dilution, and ACAT1 antibody at a 1:5000 dilution. Immunolabeling was visualized with 3,3’-diaminobenzidine (DAB) solution (zsbio, Beijing, China), followed by counterstaining with hematoxylin.

### CCK-8 assay

The CCK-8 assay was conducted using the Cell Counting Kit-8 (CCK-8) (K1018, APEBIO, Houston, USA). After 24 h of siRNA transfection, cells were seeded in 96-well plates (2 × 10³ cells per well) with 100 μL of culture medium and incubated with 10 μL of CCK-8 for 2 h. Absorbance at 450 nm was measured according to the manufacturer’s instructions. Each group was tested at 0, 24, 48, 72, and 96 h.

### Colony formation assay

Cells were seeded in 6-well culture plates at a density of 500 cells per well and incubated at 37 °C with 5% CO₂ for two weeks. After 14 days, cells were washed twice with PBS, fixed with 4% paraformaldehyde for 30 minutes, stained with 0.2% crystal violet for 30 min, washed with water, dried, and photographed.

### EdU proliferation assay

The EdU proliferation assay was conducted using the Cell-Light EdU Apollo 567 in vitro imaging kit (RiboBio, Guangzhou, China). After 24 h of siRNA transfection, cells were seeded in 96-well plates (3 × 10⁴ cells per well), incubated with 10 μM EdU for 2 hours, and then fixed with 4% paraformaldehyde. Cells were permeabilized with 0.3% Triton X-100 and stained with Apollo fluorescent dye, and nuclei were stained with DAPI. The number of EdU-positive cells was counted in three random fields using an inverted fluorescence microscope (DMI4000B, Leica, Wetzlar, Germany).

### Detection of apoptosis and reactive oxygen species (ROS)

Apoptosis was assessed using the Annexin V-FITC/PI apoptosis kit (E-CK-A211, Elascience, China) in accordance with the manufacturer’s protocol. Cells were trypsinized, washed twice with cold phosphate-buffered saline, and resuspended in 500 μL of binding buffer. Subsequently, 2.5 μL of Annexin V and 2.5 μL of PI were added, and cells were incubated in the dark for 15 minutes. After adding 400 μL of 1× Annexin V binding buffer, cells were immediately analyzed by flow cytometry, with the FITC channel used for Annexin V and the PerCP channel for PI. We used carboplatin 80 μM treatment for 24–48 h as a positive control for apoptosis. At the same time, the reactive oxygen species generation by the cells was measured with a cellular ROS detection assay kit (50101ES, YEASEN), and also analyzed by the flow cytometer. Rosup (50101ES01, YEASEN, 100 μM final concentration, 4 h incubation at 37 °C in the dark) from the Reactive Oxygen Species Assay Kit was used as the positive inducer.

### Measurement of mitochondrial membrane potential (MMP)

Changes in MMP were determined using the JC-1 detection kit (M34152, Thermo Scientific, USA). Cells were trypsinized, washed twice with cold phosphate-buffered saline, and incubated with JC-1 dye for 30 min at 37 °C. Fluorescence signals were analyzed with a 488 nm laser in the FITC and PE channels. The percentage of aggregated JC-1 was used to quantify MMP changes, indicating a decrease in MMP. CCCP (Thermo, M20036, 50 μM final concentration, 15 min incubation at 37 °C with 5% CO₂) was used to induce mitochondrial membrane depolarization as a positive control.

### LDH release assay

LDH release levels were measured using the CytoTox 96® Non-Radioactive Cytotoxicity Assay (G1780, Promega, Wisconsin, USA) according to the manufacturer’s instructions. Following experimental treatment, supernatant samples were transferred to a 96-well plate, and an equal volume of CytoTox 96® reagent was added to each well and incubated for 30 min. Absorbance was measured at 490 nm after adding the stop solution. The percentage of LDH release was calculated using the equation (LDHsample − LDHbackground)/(LDHmaximum − LDHbackground) × 100%.

### Caspase-3 activity assay

Caspase-3 activity was measured using a caspase-3 activity assay kit (C1115, Beyotime, Shanghai, China). After treatment, cells were lysed in ice-cold RIPA buffer for 30 min. Then, 50 μL of cell supernatant was added to a 96-well plate containing 40 μL of buffer and 10 μL of 2 mM caspase-3 substrate (Ac-DEVD-pNA) and incubated at 37 °C for 2 h. Caspase-3 activity was calculated by measuring optical density at 405 nm. Caspase-3 concentration in each sample was normalized to total protein.

### Liquid chromatography/Mass spectrometry (LC/MS) analysis

293 T cells expressing either vector or Flag-TRIM59 were separately constructed and treated with 20 μM MG132 (MCE, New Jersey, USA) for 6 h before harvesting. Cells were lysed in pre-cooled IP lysis buffer (87787, Thermo Scientific, USA), and after centrifugation, proteins were incubated with a Flag antibody at 4 °C overnight. The next day, A/G magnetic beads (88803, Thermo Scientific, USA) were added and incubated at room temperature for 2 h, followed by three washes with 0.5% TBST (PBS + 0.5% Tween-20) for 5 min each. The immunoprecipitate was eluted by boiling the beads directly in 1× loading buffer. Samples were separated by SDS-PAGE and stained with Coomassie Brilliant Blue. Cell pellets were shipped to Novogene Co., Ltd. for LC/MS and data analysis.

### Co-immunoprecipitation

Cells were treated with 20 μM MG132 for 6 h prior to collection. After protein extraction and quantification, 5 mg of protein and 5 μg of primary antibody or IgG were incubated at 4 °C overnight. The next day, 50 μL of protein A/G magnetic beads were added to each sample and incubated at room temperature for 2 h. After elution, immune complexes were subjected to Western blot analysis.

### Cycloheximide (CHX) chase assay

Cycloheximide (S7418, Sigma, USA) was resuspended in DMSO (200 mM) and stored at −20 °C before use. Cells were seeded in 6-well plates in 2 mL of culture medium and incubated with 50 μg/mL CHX at 37 °C with 5% CO₂ for various time points. Cells were then collected for further analysis by Western blot.

### Metabolomics and data analysis

ACHN cells with stable ACAT1 expression and TRIM59 knockdown were constructed and cultured six times. Cells were washed three times with cold PBS, scraped with a cell scraper, and collected in EP tubes. After centrifugation, the supernatant was discarded, and 1 mL of pre-cooled methanol was added. EP tubes were placed in liquid nitrogen for 5 min. Cell pellets were sent to Shanghai Applied Protein Technology Co. Ltd. for absolute quantitative non-targeted lipid metabolomics and data analysis.

### Enzyme-linked immunosorbent assay

At the end of the cell culture, the medium was collected and centrifuged at 3000 rpm for 3 minutes to remove cell debris. Cells were washed with cold PBS and lysed in lysis buffer, with total protein quantified in each well. For tissue homogenates, tissues were rinsed with pre-cooled PBS (0.01 M, pH = 7.4) to remove blood residues, minced after weighing, and combined with PBS in a glass homogenizer. Homogenates were centrifuged at 4 °C, 5000 g for 5 min, and the supernatant was collected for analysis. Cardiolipin concentration in cell culture or tissue was measured using a human cardiolipin ELISA kit (MM-2281H1, Meimian, Jiangsu, China) per the manufacturer’s instructions. Cardiolipin concentration was normalized to total protein.

### Animal experiments

Six-week-old female BALB/c mice were purchased from GemPharmatech (Jiangsu, China) and housed in the SPF animal facility of the Experimental Animal Resource Center of Sun Yat-sen University under controlled conditions (12 h light/12 h dark cycle). All procedures involving animals were approved by the Animal Ethics Committee of Sun Yat-sen University (approval number: 2022002554). Female BALB/c mice at six weeks of age were randomly divided into five groups, each group with 6 mice. A total of 1 × 10⁷ ACHN cells were subcutaneously injected into each BALB/c mouse. After xenografts became palpable, mice were randomly assigned to different groups. Each group received multiple injections of TRIM59 siRNA (5 nmol, 0.1 mL) three times per week for 39 days in and around the tumor. Tumor volume was measured every 3 days with calipers and calculated using the formula length × width². After 39 days, mice were sacrificed, and tumors and harvested organs were subjected to IHC staining and ELISA. The maximal tumor size permitted by the ethics committee was 1000 mm³, and no xenograft in this study exceeded this limit. All investigators involved in tumor cell inoculation, drug administration, and outcome assessment were blinded to group allocation throughout the study.

### Statistical analysis

Statistical analyses were performed using GraphPad Prism 9.5 software (CA, USA). Experimental data were expressed as mean ± s.d. from three independent experiments. Data were analyzed by two-tailed unpaired Student’s t test between two groups and by one-way ANOVA for multiple comparison. Spearman’s correlation was performed to assess the relationship between TRIM59 expression and ACAT1 expression. *P* < 0.05 considered statistically significant.

## Supplementary information

Supplemental data

Original data

Original data

Original data

Original data

Original data

Original data

Original data

## Data Availability

The datasets used and/or analyzed during the current study are available from the corresponding authors (clarelynn_lin@163.com) upon reasonable request.
