# Supplementary material for: TRIM59 suppresses mitochondrial-associated apoptosis to facilitate progression in papillary renal cell carcinoma via the ACAT1-cardiolipin pathway
Source: Cell Death Dis. 2025 Aug 11;16(1):606. doi: 10.1038/s41419-025-07913-5 (PMC12339706; doi:10.1038/s41419-025-07913-5)
Supplement: Supplementary file 1 — Supplemental data [file 41419_2025_7913_MOESM1_ESM.pdf]

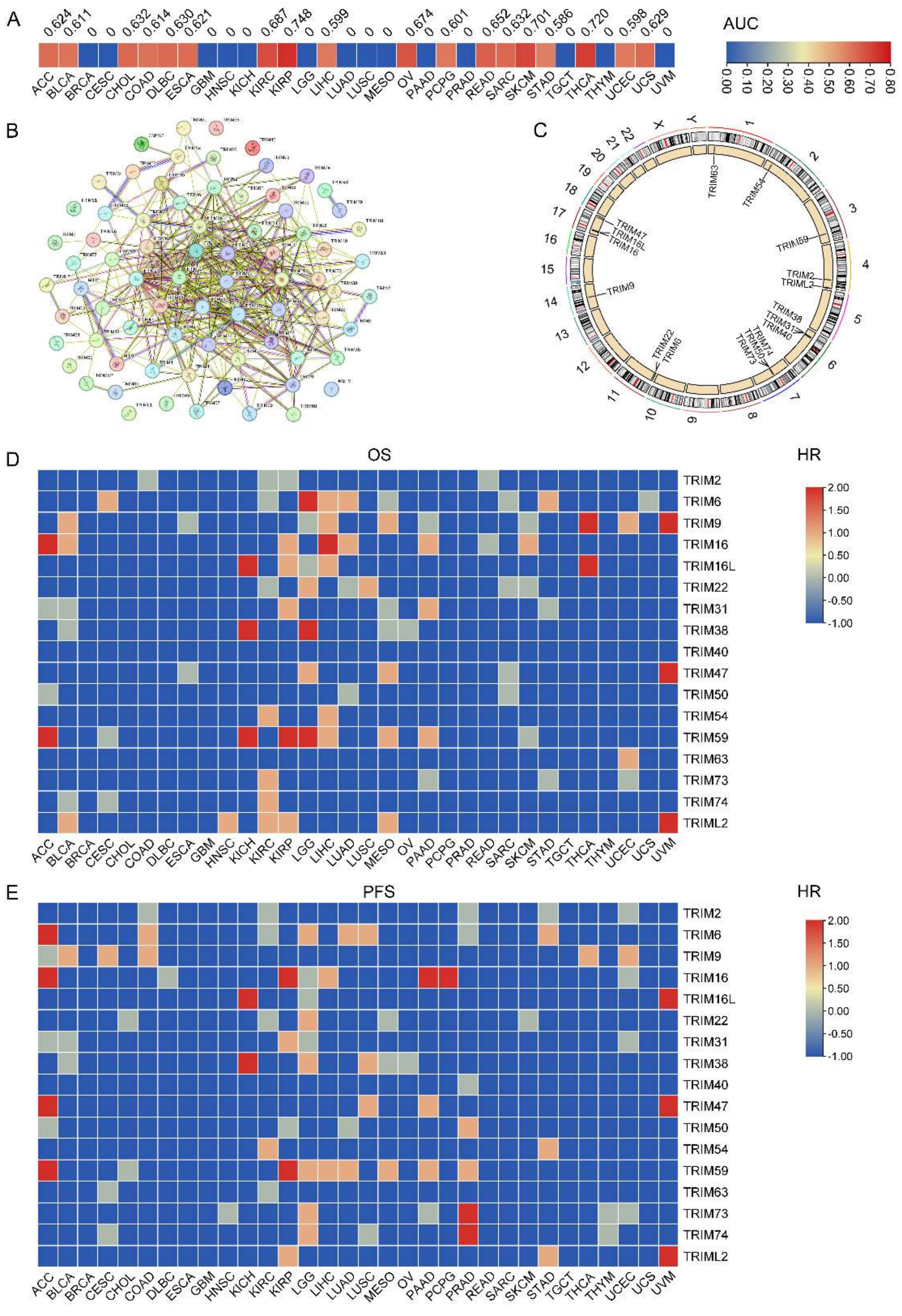

Figure S1: The interaction of TRIM family in pan carcinoma and its importance for clinical treatment prediction.

(A) AUC values of TRIM model in 32 tumors of TCGA.

(B) TRIM family genes interact at the protein level. The connection between nodes represents the interaction between two proteins, and different colors correspond to different types of interactions.

(C) The locations of copy number variation in DMEs of 17 TRIM human chromosome pairs.

(D) The correlation between the expression levels of TRIM gene family and OS HR in pan cancer datasets.

(E) The correlation between the expression levels of TRIM gene family and PFS HR in pan cancer datasets.

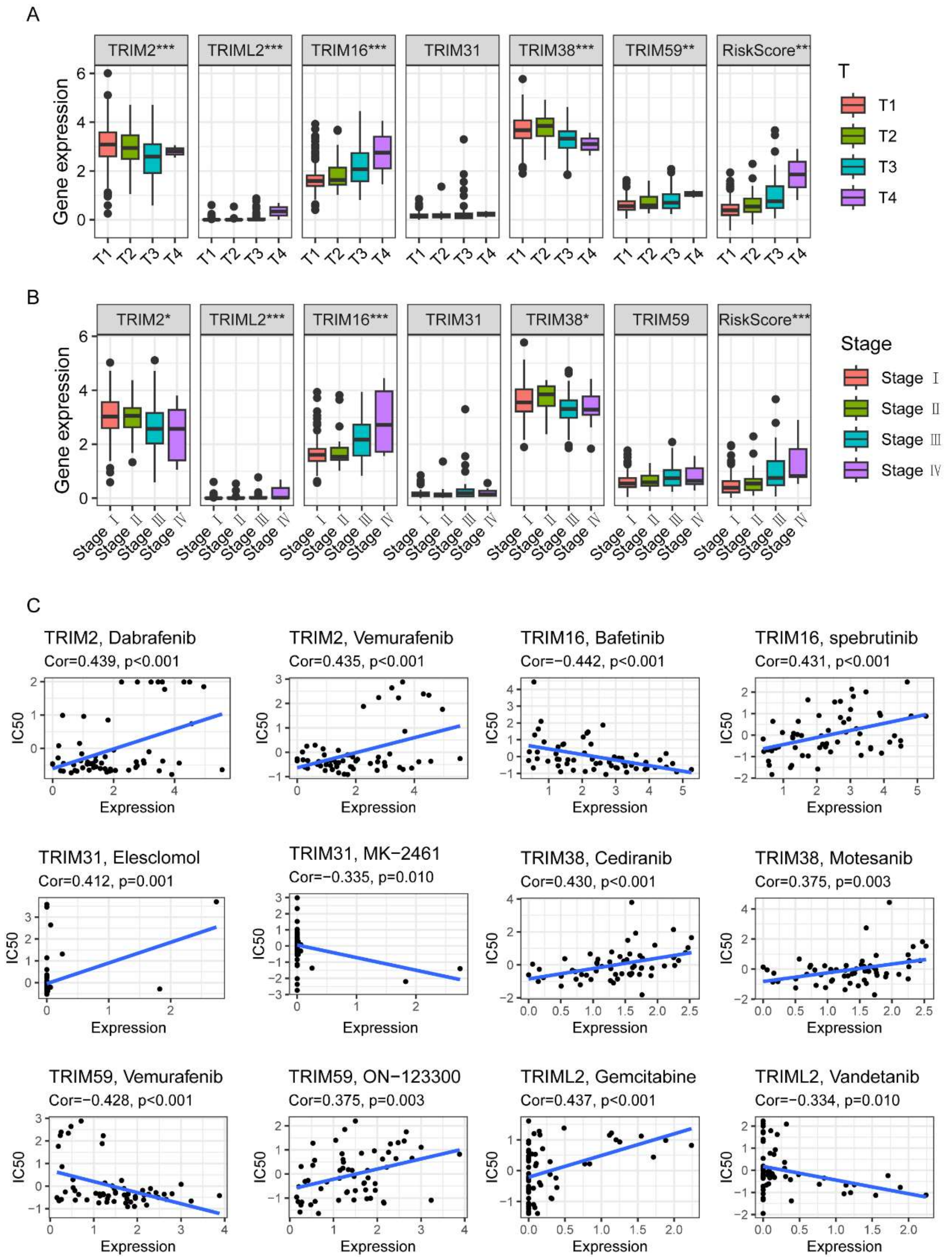

Figure S2: The relationship between RiskScore of TRIM family gene expression and clinical stage and drug sensitivity.

(A) The expression of TRIM family genes in different T stages of KIRP of TCGA.

(B) The expression of TRIM family genes in different clinical stages of KIRP of TCGA.

(C) The relationship between RiskScore of TRIM family gene expression levels and drug sensitivity in pan cancer datasets. The abscissa represents gene expression, and the ordinate represents drug sensitivity.

*P < 0.05; **P < 0.01; ***P < 0.001.

A

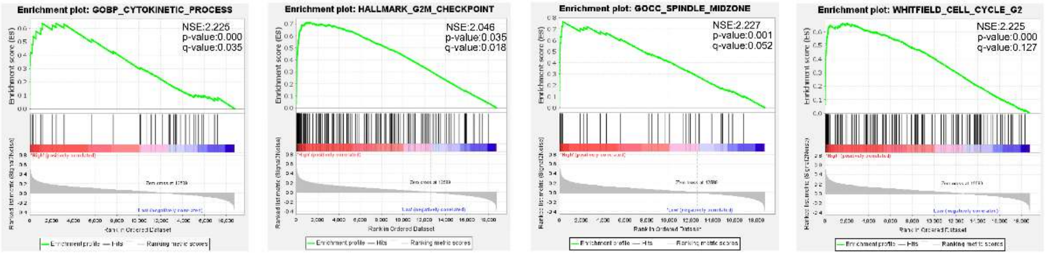

Figure S3: GSEA analysis based on the median value of RiskScore.

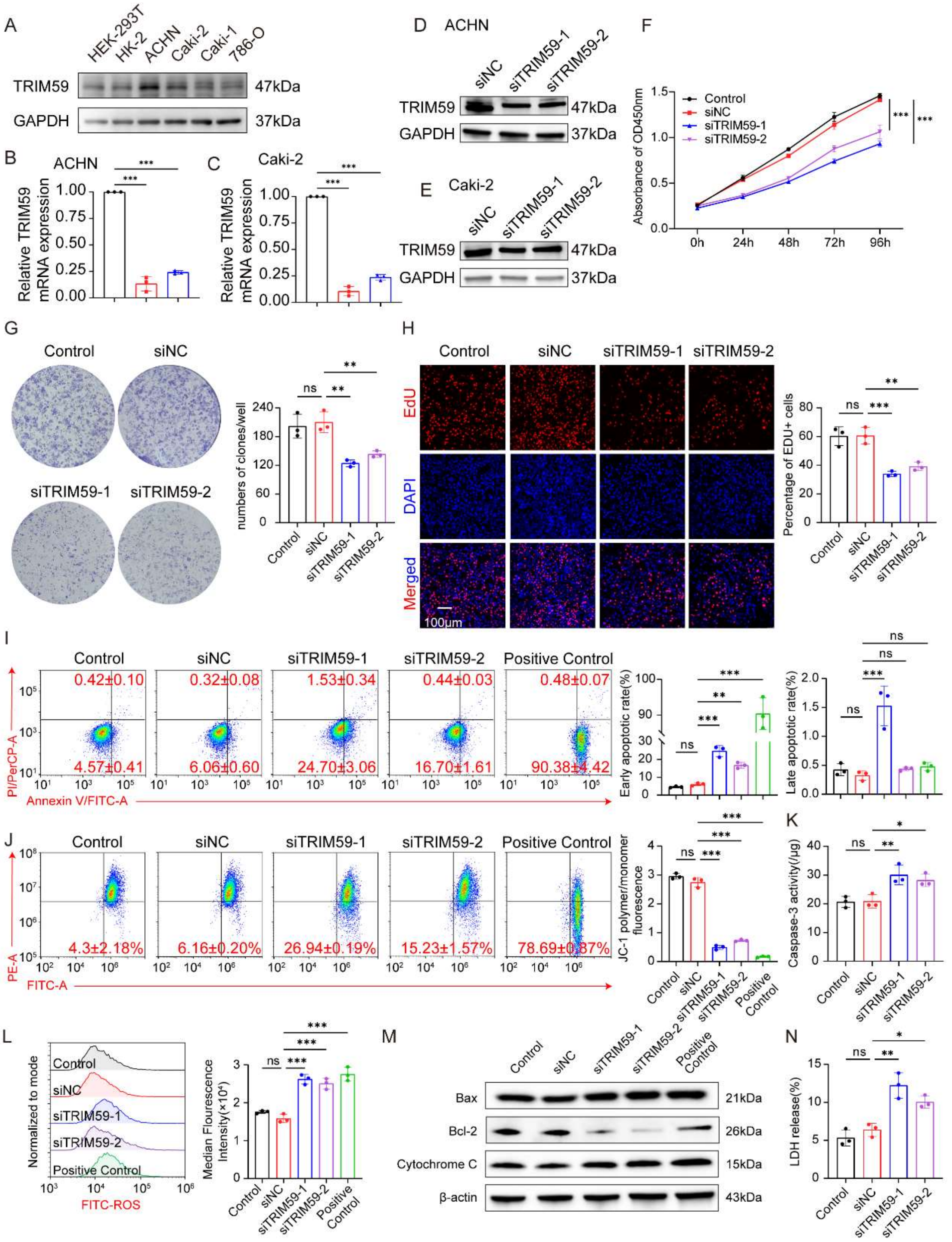

Figure S4: TRIM59 suppresses the mitochondria related apoptosis of papillary renal cell carcinoma.in Caki-2 cells.

(A) Western blot analysis was used to detect the TRIM59 protein expression in HEK-293T, HK-2, ACHN, Caki-2, Caki-1 cells, and 786-O cells.

(B, C) Detection of TRIM59 knockdown efficiency by qPCR in ACHN cells (B) and Caki-2 cells (C).

(D, E) Detection of TRIM59 knockdown efficiency by Western blot in ACHN cells (D) and Caki-2 cells (E).

(F, G, H) TRIM59 silencing contributed to attenuating tumorigenesis, which was evaluated by CCK-8 assay (F), colony formation assay (G) and EdU (H) staining to detect the proliferation of Caki-2 cells, and the quantitative analysis of colony formation assay and EdU staining in Caki-2 cells.

(I) Cell apoptosis followed by TRIM59 silencing was assayed via Annexin V/PI double staining in Caki-2 cells, and the quantitative analysis of the ratio of the apoptotic cells. (Positive Control: carboplatin 80 μM for 48h)

(J) TRIM59 silencing reduced the mitochondrial membrane potential (MMP) in Caki-2 cells. MMP was measured by flow cytometry using JC-1 staining. Red fluorescence (PE channel) represented polymers and green fluorescence (FITC channel) represented monomers. (Positive Control: CCCP 50 μM for 15 minutes)

(K) Caspase-3 activity assay was utilized to determine apoptosis after knocking-down TRIM59 in Caki-2 cells.

(L) Flow cytometry (left) and statistical analysis (right) show the ROS levels after TRIM59 knockdown in Caki-2 cells. (Positive Control: Rosup 100 μM for 4h)

(M) WB detection of expression levels of Bax, Bcl-2, Cytochrome C, and β-actin after TRIM59 knockdown in Caki-2 cells. (Positive Control: carboplatin 80 μM for 48h)

(N) LDH assay was utilized to determine apoptosis after knocking-down TRIM59 in Caki-2 cells.

The experiments were repeated 3 times independently. Data are presented as the mean ± s.d..

*P < 0.05; **P < 0.01; ***P < 0.001.

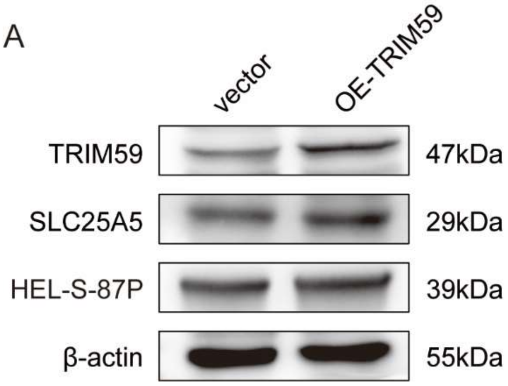

Figure S5: Western blot analysis indicated that TRIM59 does not regulate the expression of SLC25A5 and HEL-S-87P.

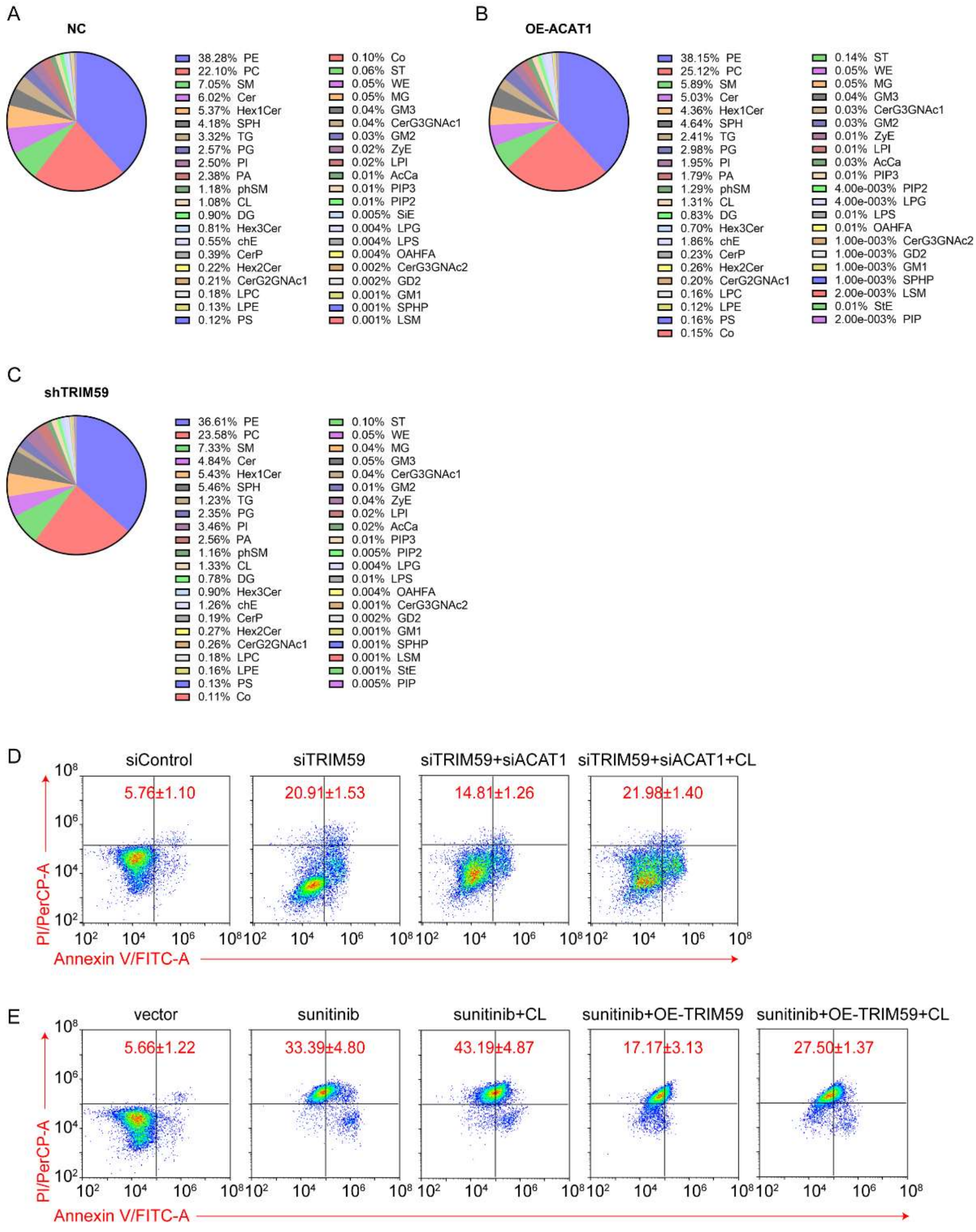

Figure S6: Changes in the proportion of lipid metabolism in each group and the importance of cardiolipin in TRIM59-mediated acat1 inhibition of apoptosis. (A, B, C) The pie charts illustrated the proportions of different lipid subclasses in the NC group, shTRIM59 group, and OE-ACAT1 group.

(D) Apoptotic effect of TRIM59 silencing, ACAT1 silencing, and cardiolipin 50 μM for 6h revealed via Annexin V/PI double staining in ACHN cells.

(E) Apoptotic effect of TRIM59 overexpressing, sunitinib 5 μM for 6h, and cardiolipin 50 μM for 6h revealed via Annexin V/PI double staining in ACHN cells.

The experiments were repeated 3 times independently. Data are presented as the mean ± s.d..
