# Supplementary figures and images for: TRIM59 suppresses mitochondrial-associated apoptosis to facilitate progression in papillary renal cell carcinoma via the ACAT1-cardiolipin pathway
Source: Cell Death Dis. 2025 Aug 11;16(1):606. doi: 10.1038/s41419-025-07913-5 (PMC12339706; doi:10.1038/s41419-025-07913-5)

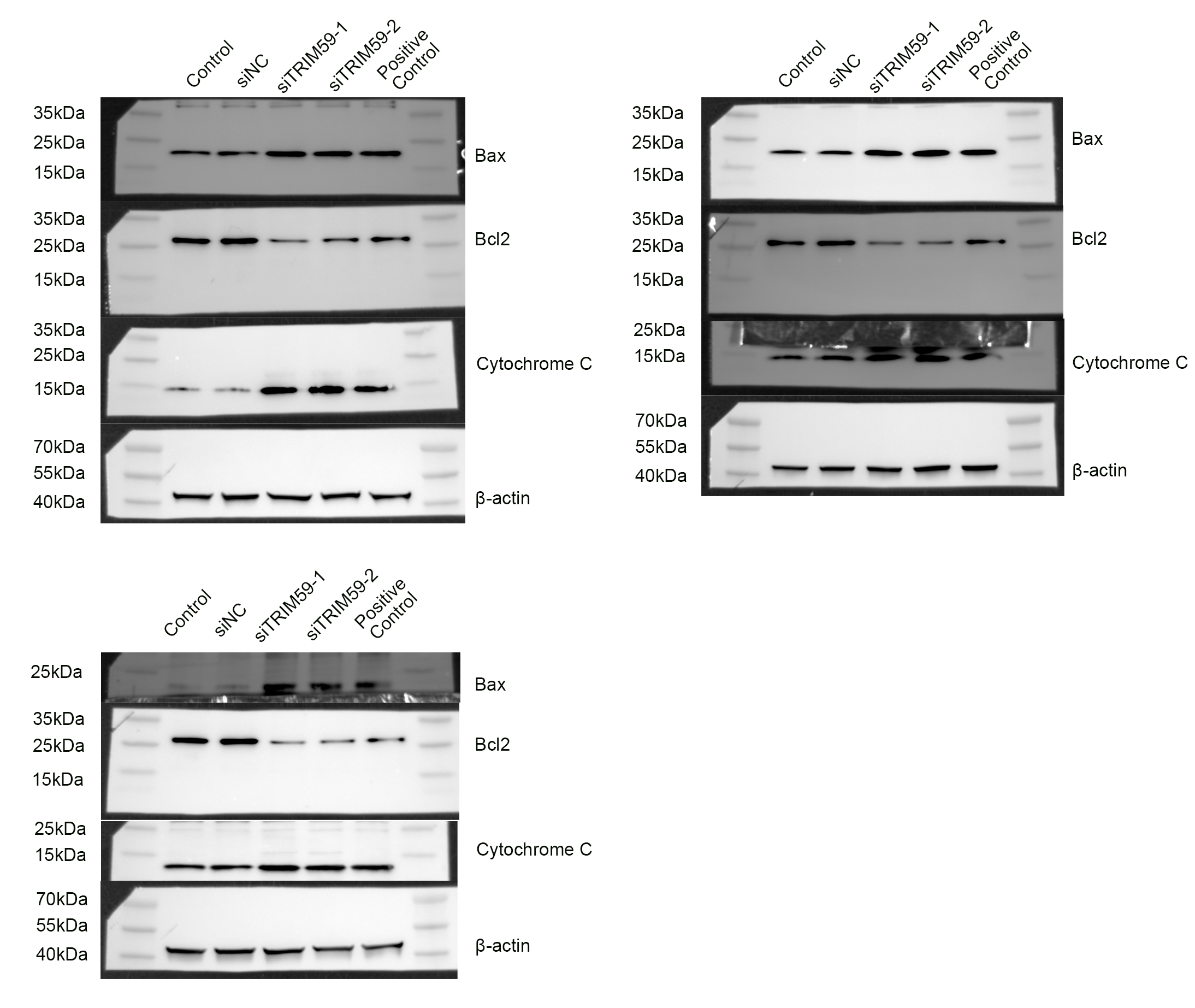

Supplement: Supplementary file 2 — Original data [file 41419_2025_7913_MOESM2_ESM.png]

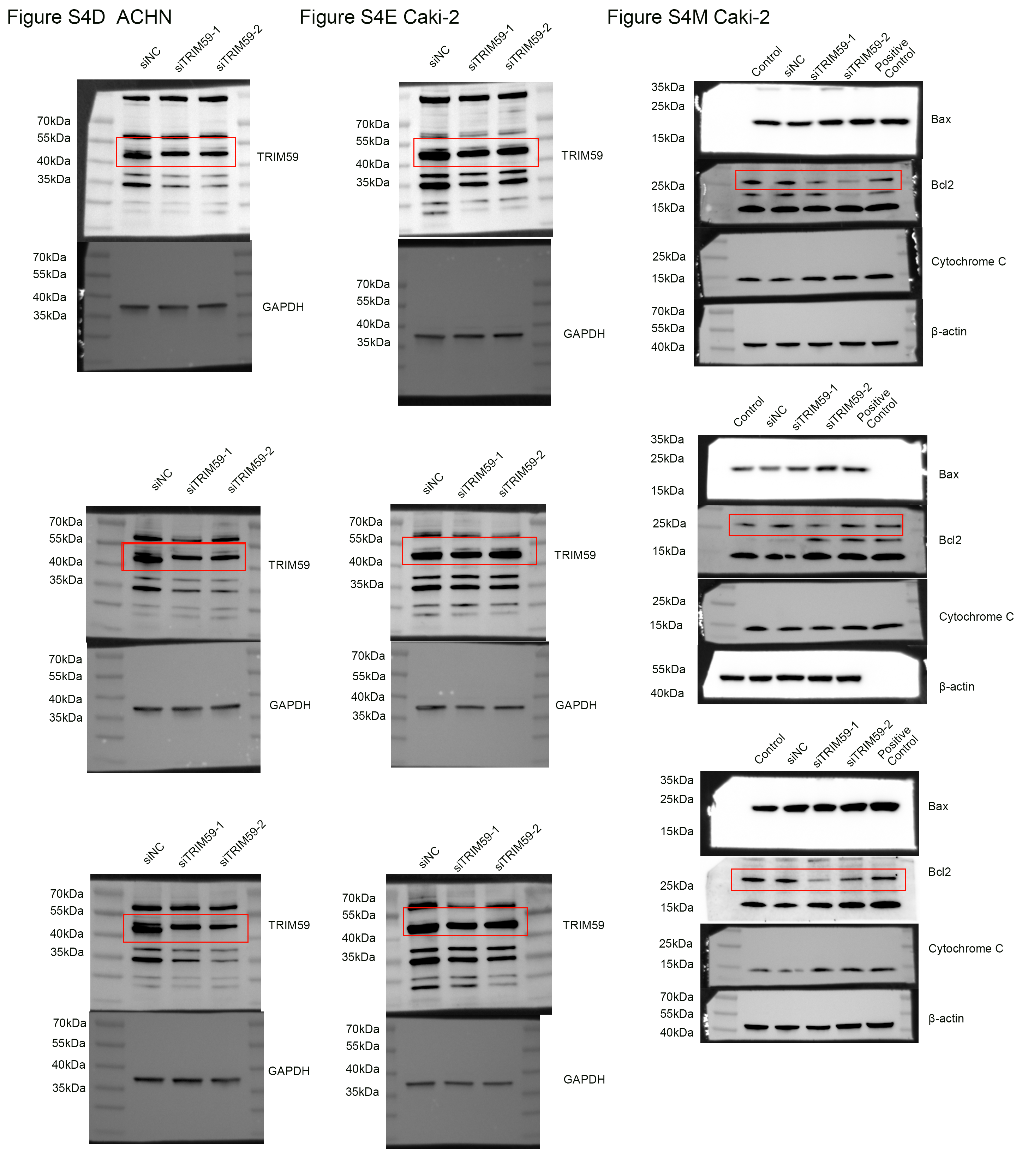

Supplement: Supplementary file 3 — Original data [file 41419_2025_7913_MOESM3_ESM.png]

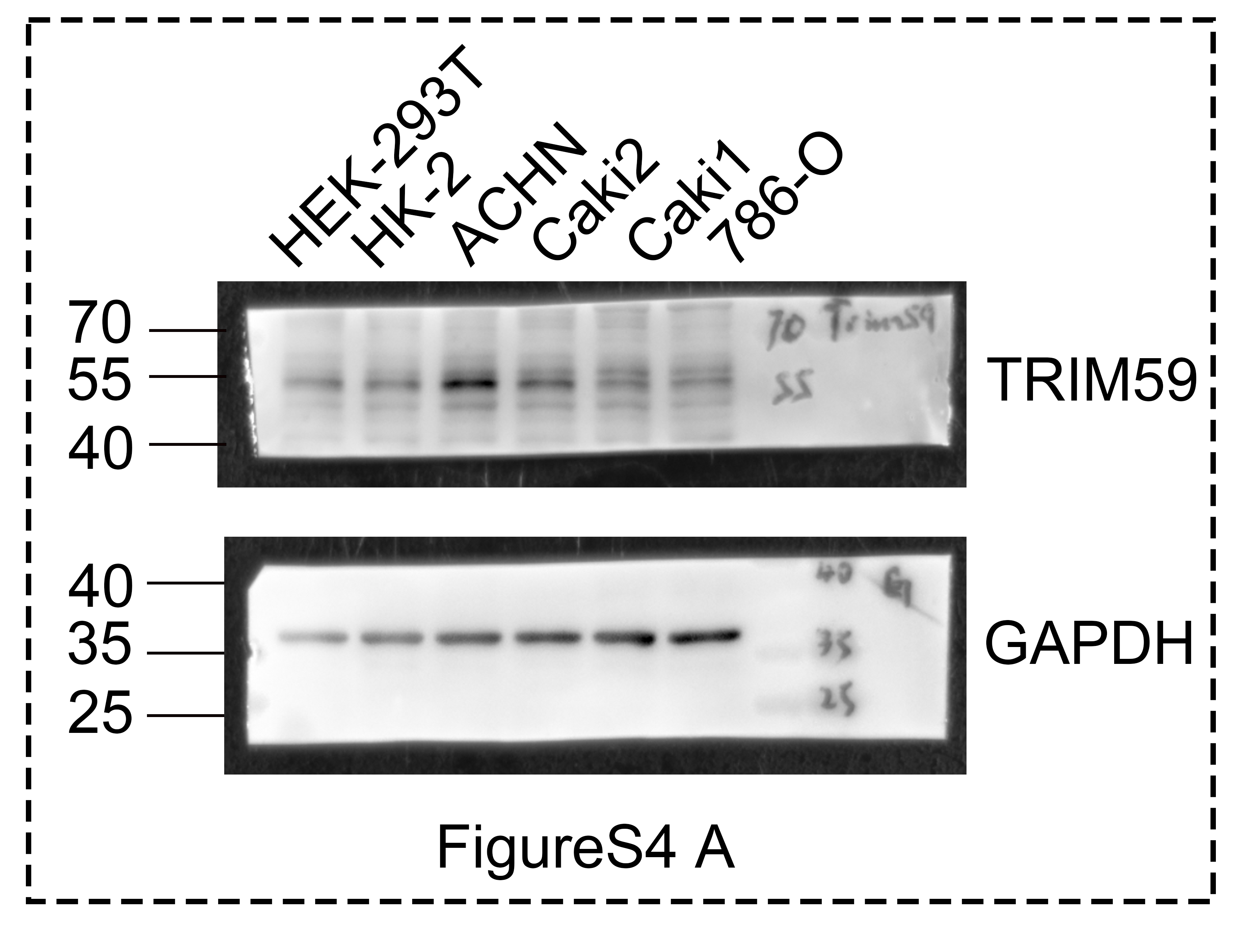

Supplement: Supplementary file 4 — Original data [file 41419_2025_7913_MOESM4_ESM.png]

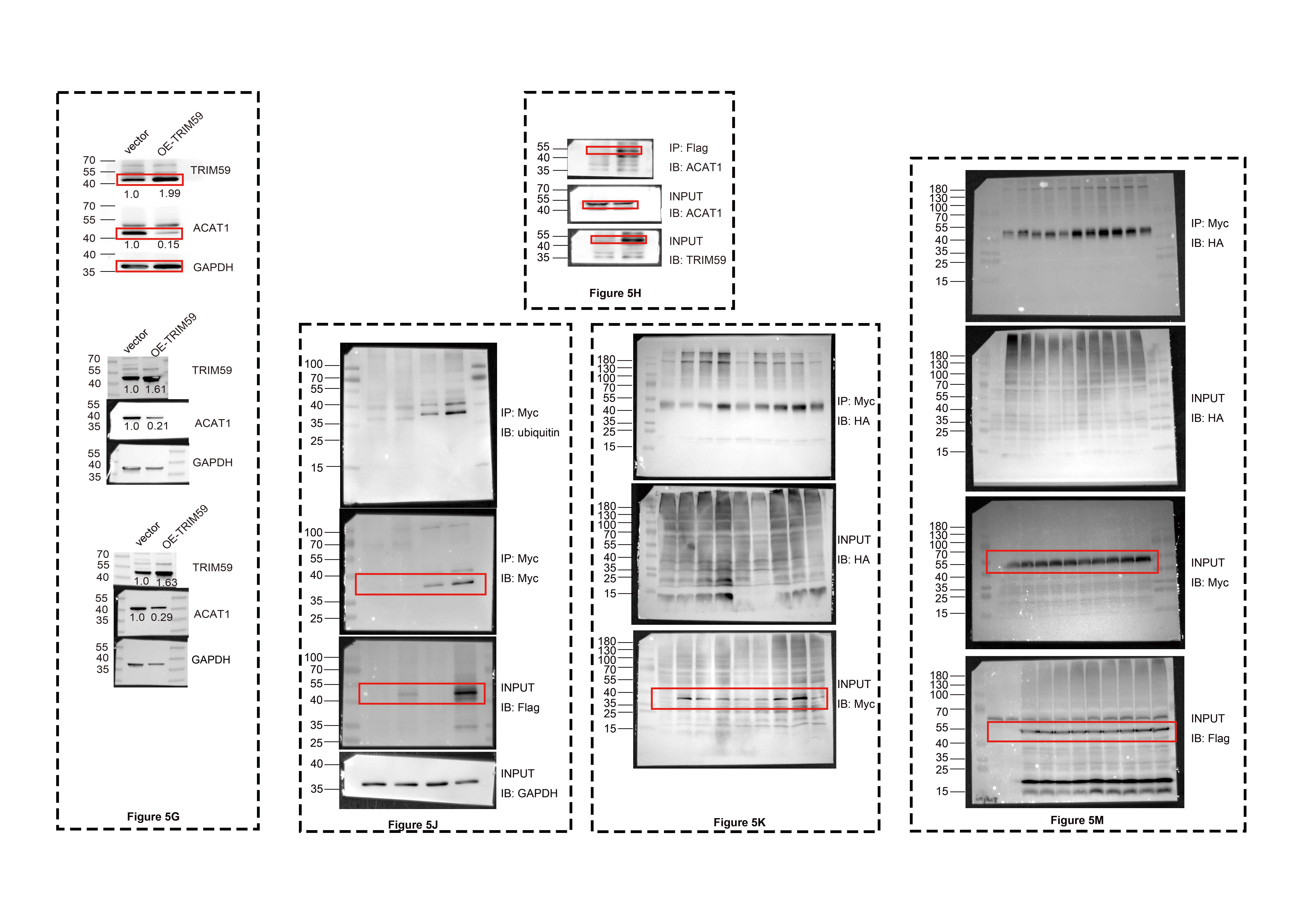

Supplement: Supplementary file 5 — Original data [file 41419_2025_7913_MOESM5_ESM.png]

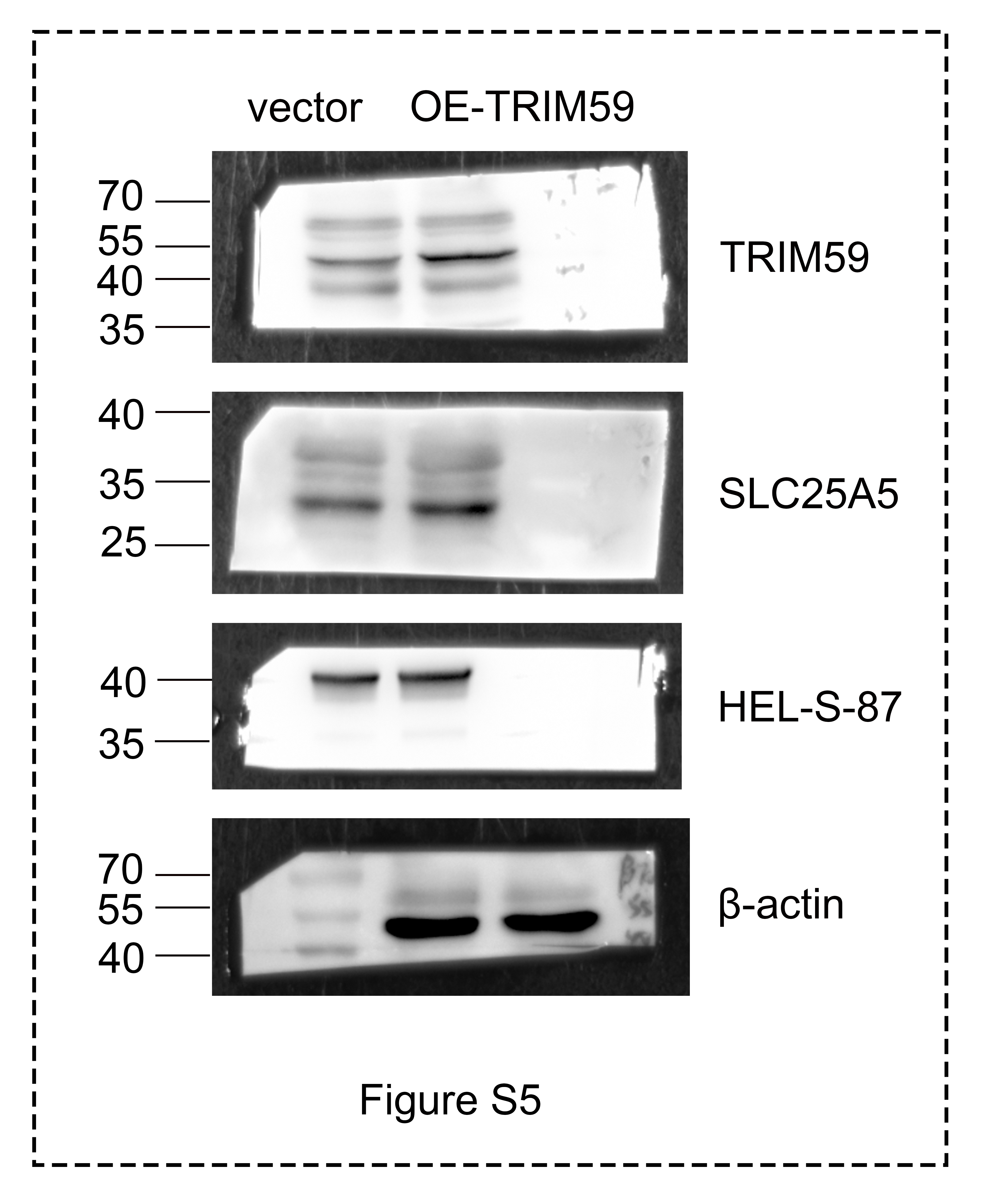

Supplement: Supplementary file 6 — Original data [file 41419_2025_7913_MOESM6_ESM.png]

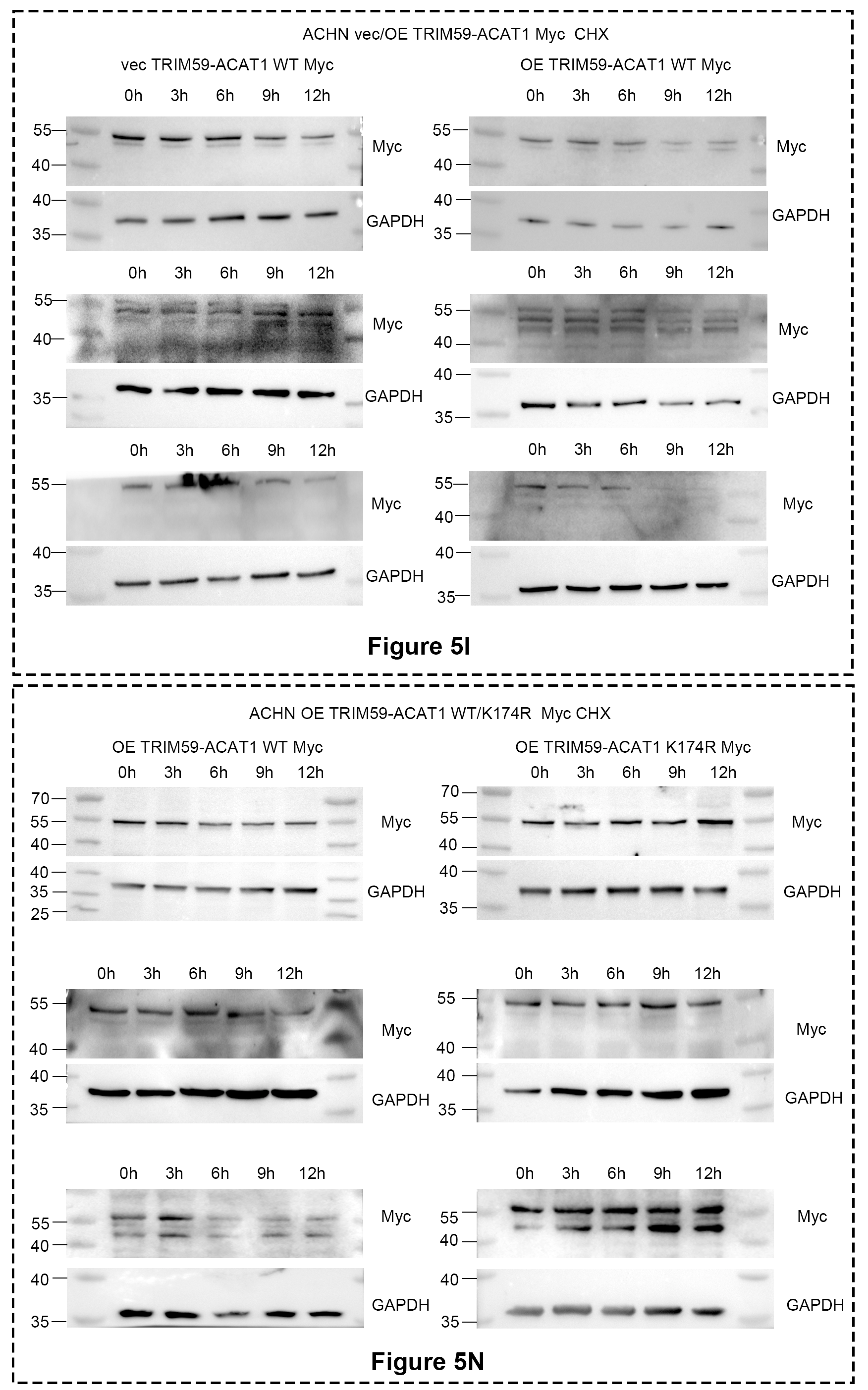

Supplement: Supplementary file 7 — Original data [file 41419_2025_7913_MOESM7_ESM.tif]

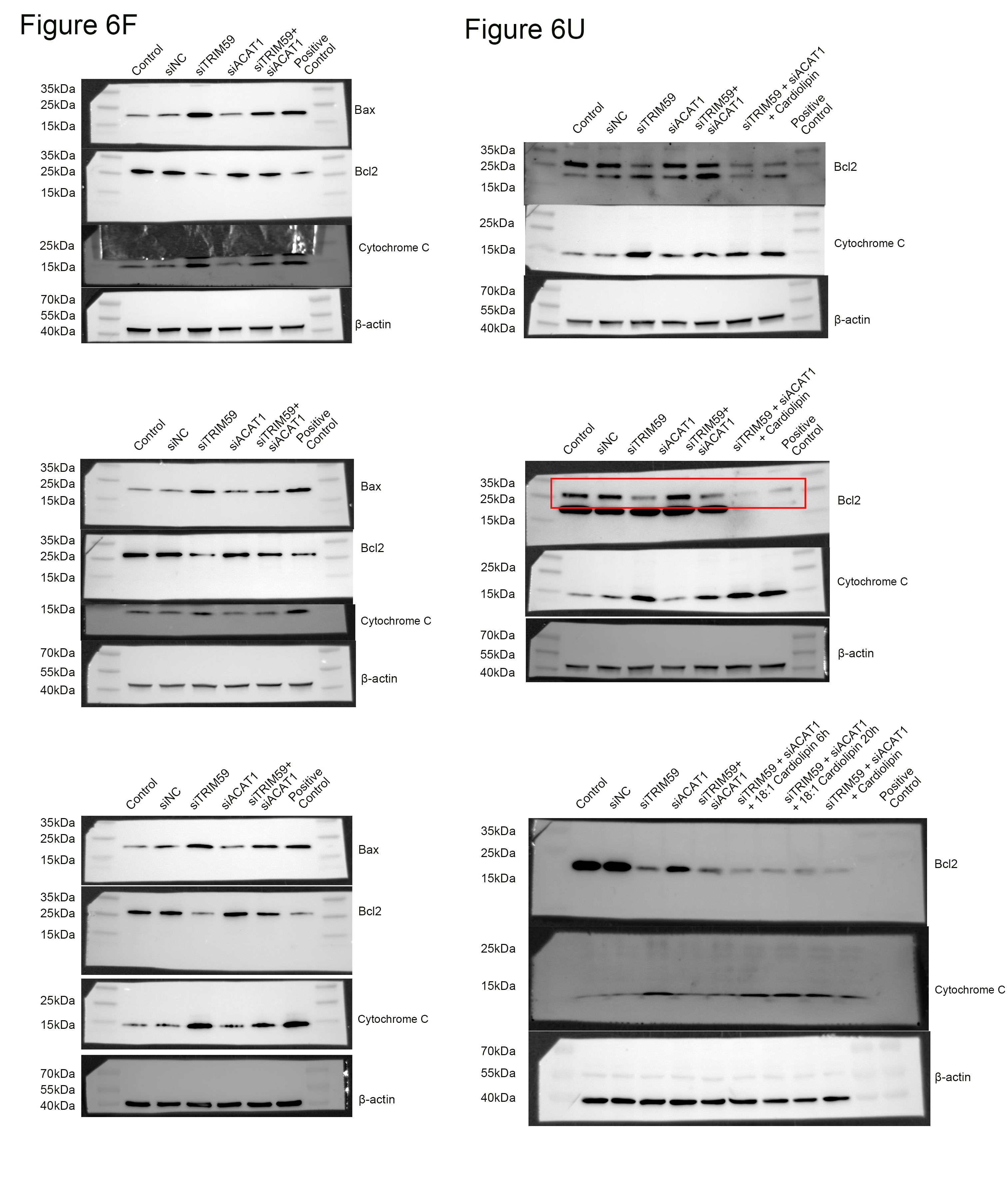

Supplement: Supplementary file 8 — Original data [file 41419_2025_7913_MOESM8_ESM.png]
